# Characterization of the Role of Two-Component Systems in Antibiotic Resistance Formation in Salmonella enterica Serovar Enteritidis

**DOI:** 10.1128/msphere.00383-22

**Published:** 2022-10-26

**Authors:** Mengjun Hu, Xiaozhen Huang, Xuebin Xu, Zengfeng Zhang, Shoukui He, Jinyu Zhu, Hong Liu, Xianming Shi

**Affiliations:** a Department of Food Science & Technology, School of Agriculture & Biology, Shanghai Jiao Tong University, Shanghai, China; b State Key Lab of Microbial Metabolism, Shanghai Jiao Tong University, Shanghai, China; c Shanghai Municipal Center for Disease Control and Prevention, Shanghai, China; University of Rochester

**Keywords:** *S*. Enteritidis, two-component system, multidrug resistance, GlnGL, CpxAR, PhoPQ

## Abstract

The two-component system (TCS) is one of the primary pathways by which bacteria adapt to environmental stresses such as antibiotics. This study aimed to systematically explore the role of TCSs in the development of multidrug resistance (MDR) in Salmonella enterica serovar Enteritidis. Twenty-six in-frame deletion mutants of TCSs were generated from *S*. Enteritidis SJTUF12367 (the wild type [WT]). Antimicrobial susceptibility tests with these mutants revealed that 10 TCSs were involved in the development of antibiotic resistance in *S*. Enteritidis. In these 10 pairs of TCSs, functional defects in CpxAR, PhoPQ, and GlnGL in various *S*. Enteritidis isolates led to a frequent decrease in MIC values against at least three classes of clinically important antibiotics, including cephalosporins and quinolones, which indicated the importance of these TCSs to the formation of MDR. Interaction network analysis via STRING revealed that the genes *cpxA*, *cpxR*, *phoP*, and *phoQ* played important roles in the direct interaction with global regulatory genes and the relevant genes of efflux pumps and outer membrane porins. Quantitative reverse transcription-PCR analysis further demonstrated that the increased susceptibility to cephalosporins and quinolones in Δ*phoP* and Δ*cpxR* mutant cells was accompanied by increased expression of membrane porin genes (*ompC*, *ompD*, and *ompF*) and reduced expression of efflux pump genes (*acrA*, *macB*, and *mdtK*), as well as an adverse transcription of the global regulatory genes (*ramA* and *crp*). These results indicated that CpxAR and PhoPQ played an important role in the development of MDR in *S*. Enteritidis through regulation of cell membrane permeability and efflux pump activity.

**IMPORTANCE** *S*. Enteritidis is a predominant Salmonella serotype that causes human salmonellosis and frequently exhibits high-level resistance to commonly used antibiotics, including cephalosporins and quinolones. Although TCSs are known as regulators for bacterial adaptation to stressful conditions, which modulates β-lactam resistance in Vibrio parahaemolyticus and colistin resistance in Salmonella enterica serovar Typhimurium, there is little knowledge of their functional mechanisms underlying the development of antibiotic resistance in *S*. Enteritidis. Here, we systematically identified the TCS elements in *S*. Enteritidis SJTUF12367, revealed that the three TCSs CpxAR, PhoPQ, and GlnGL were crucial for the MDR formation in *S*. Enteritidis, and preliminarily illustrated the regulatory functions of CpxAR and PhoPQ for antimicrobial resistance genes. Our work provides the basis to understand the important TCSs that regulate formation of antibiotic resistance in *S*. Enteritidis.

## INTRODUCTION

Salmonella enterica serovar Enteritidis is an important foodborne pathogen causing diarrhea, fever, and abdominal cramps in humans and animals worldwide, and its multidrug resistance (MDR) is of significant global concern (1). Among MDR isolates, the ACSSuT resistance pattern (defined as resistance to ampicillin, chloramphenicol, streptomycin [STR], sulfamethoxazole, and tetracycline [TET]) is a common characteristic in Salmonella infection (2, 3). Quinolones and cephalosporins are currently recommended for treatment of bacterial gastrointestinal diseases clinically (4). However, *S*. Enteritidis isolates carrying the ACSSuT profile and simultaneously coresistant to quinolones and cephalosporins have emerged, which can complicate clinical therapy. A relatively high incidence of resistance to nalidixic acid (NAL) (95.29%), cefotaxime (CTX) (70.64%), cefepime (FEP) (58.72%) and ceftazidime (CAZ) (48.62%) has been reported among *S*. Enteritidis isolates with the ACSSuT profile derived from patients (5, 6). Therefore, it is of critical importance to understand the molecular mechanisms underlying the emergence of MDR and to develop therapeutic alternatives for these problematic *S*. Enteritidis strains.

MDR in many cases may result from potential synergies, including reduced outer membrane (OM) permeability, degraded antimicrobial agents, an altered drug target, and activated drug export. The genetic basis for resistance to β-lactam antibiotics (e.g., penicillin and extended-spectrum cephalosporins) is usually due to intrinsic or horizontally acquired β-lactamases and multiple drug exporters that can degrade and efflux commonly prescribed antibiotics (7). In addition, mutations in DNA gyrase (GyrA and GyrB) and topoisomerase IV (ParC and ParE) and activation of efflux pumps (e.g., AcrAB) are usually responsible for high-level quinolone resistance (8). Moreover, the genes responsible for outer membrane (OM) porins (e.g., OmpC and OmpF), which act as checkpoints to monitor the nonspecific entry of many compounds, could impact membrane permeability and therefore promote an increase in tolerance to quinolones and cephalosporins in Salmonella (9, 10). Previous studies have mainly focused on the number and types of antimicrobial resistance genes (ARGs), while their modes of activation and regulation by stress response regulators such as two-component systems (TCSs) in the presence of antibiotics are rarely characterized and need to be clarified.

TCSs are crucial signal transduction system composed of a sensor histidine kinase (HK) and a response regulator (RR), by which bacteria sense and respond to environmental stresses (e.g., temperature, pH, osmolarity, and antibiotics) (11, 12). Typically, HKs sense environmental signals, autophosphorylate at the conserved histidine residue, and subsequently transfer phosphoryl to its cognate cytosolic RR at the conserved aspartate residue. The activated RR then binds to specific gene promoters to regulate their expression and therefore initiates cellular responses (11). Bacteria usually possess various TCSs, ranging from a few to over 100, and certain TCSs have been shown to contribute to antibiotic resistance formation via the regulation of ARG expression (13). For example, VbrKR in Vibrio parahaemolyticus directly senses β-lactam antibiotics and induces the expression of *blaA*, which encodes a functional β-lactamase to destroy or hydrolyze β-lactam antibiotics (12). EvgSA serves as the master regulator to control the expression of the drug efflux genes *emrKY*, *yhiUV*, *acrAB*, and *mdfA*, thus conferring MDR to Escherichia coli (14). BlrAB in *Aeromonas* spp. and CreBC in E. coli and Pseudomonas aeruginosa trigger the expression of *ampC* (which encodes β-lactamase) and thus give rise to β-lactam resistance (15–17). Therefore, interpretation of the relationship between TCSs and ARGs is of great significance to elucidate the antibiotic resistance regulatory mechanism.

The effect of TCSs on resistance formation has seldomly determined in Salmonella, and only a few reports have shown TCSs conferring antibiotic resistance in Salmonella enterica serovar Typhimurium. For instance, it is reported that CpxAR confers resistance to aminoglycosides and β-lactams by influencing the expression level of the MDR-related genes (e.g., *acrB* and *marA*) in *S.* Typhimurium (18). PhoPQ regulates the lipopolysaccharide (LPS) modification loci (e.g., *pbgP* and *ugd*) and activates the TCS BasSR, which is responsible for LPS synthesis and lipid A modification to increase colistin (COL) resistance of *S.* Typhimurium (19). However, these studies are limited to clarifying the function of a certain TCS with susceptible S. *Typhimurium* isolates. Whether these regulatory mechanisms still work or not in common MDR isolates remains unknown. In addition, the TCSs CpxAR and PhoPQ are broadly conserved among many pathogenic and nonpathogenic bacteria, but their regulons vary in different bacteria, such as *S.* Typhimurium, E. coli, P. aeruginosa, Yersinia pestis, and Mycobacterium tuberculosis (20–24). Previous comparative genomic studies have suggested that regulons exhibit considerable plasticity across the evolution of bacterial species (25, 26). In this regard, it has more practical significance to systematically study the contribution of TCSs, including CpxAR and PhoPQ, in MDR *S*. Enteritidis.

To date, most isolates of MDR *S*. Enteritidis from both food and clinical settings exhibit high resistance to clinically recommended antibiotics (e.g., quinolones and cephalosporins), thus limiting the clinic treatment options. Anti-signal transduction is an alternative therapy to defeat antibiotic-resistant bacteria, and thus TCSs are considered attractive antibacterial targets (27). Therefore, the systematic identification and elucidation of TCSs that contribute to MDR are crucial for the prevention and control of antibiotic resistance in *S*. Enteritidis. We have recently reported the complete genome of a sequence type 11 (ST11) *S*. Enteritidis isolate, SJTUF12367, with an extensive antibiotic resistance profile and abundant ARGs (28). *S*. Enteritidis ST11 strains have been reported to be a globally dominant clone, in which MDR phenotypes are common (29). The purpose of the present work was to systematically characterize the role of the TCSs in development of MDR in *S*. Enteritidis SJTUF12367 and other related clinical isolates through gene knockout and antimicrobial susceptibility tests and to explore the regulatory mechanisms of two important TCSs (CpxAR and PhoPQ) against quinolones and cephalosporins through interaction network analysis, quantitative reverse transcription-PCR (qRT-PCR), and membrane perturbation analysis. This work will contribute to a better understanding of antibiotic resistance mechanisms as well as a basis for combating antibiotic resistance in S. Enteritidis.

## RESULTS

### Functional defects in 10 pairs of TCSs resulted in an increase in *S.* Enteritidis susceptibility to antibiotics.

**FIG 1:**
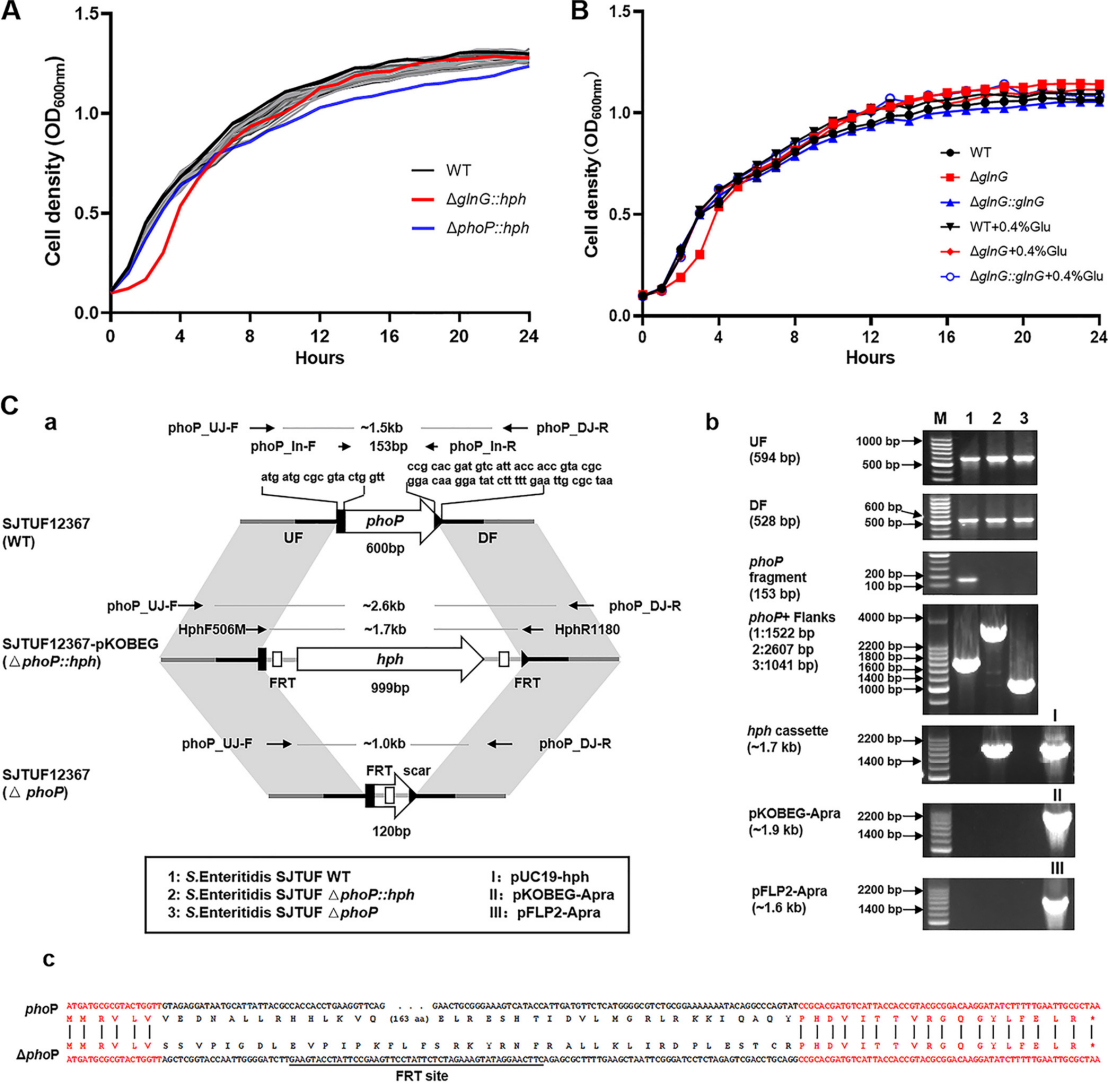
Construction of TCS mutants and determination of bacterial growth curves. (A) Comparison of growth curve in the WT and mutants. Cell densities were measured as OD_600_ every 30 min for 24 h. Growth curves represent the average of 3 biological replicates. (B) Bacterial growth of WT, Δ*glnG*, and Δ*glnG*::*glnG* strains in LB medium or LB supplemented with 0.4% glutamine (Glu); (C) schematics of *phoP* deletion and confirmation. (a) Schematics of *phoP* deletion. Black lines and parts in the gene indicate the upstream flank (UF) and downstream flank (DF). For in-frame mutagenesis, the mutation maintained a fraction of the codons of the gene. Thin arrows with names above each sequence indicate primers (Table S5) targeting corresponding positions of the sequence (not to scale). Amplicon sizes are shown in between for each pair of primers. (b) Mutant confirmation by PCR. The primers used to check *phoP*, *phoP* plus flanking sequences, and the *hph* cassette are shown in panel a. Primers EBGNHe-5/EBGh3-3 and PR1655/PR1656 were used for determination of pKOBEG-Apra and pFLP2-Apra, respectively. Expected amplicon sizes are given in parentheses. (c) Mutations confirmed by sequencing. Only the coding sequences are shown. Red text indicates the maintained codons and the corresponding translation of amino acids.

Twenty-six RR genes were replaced by a flippase recognition target (FRT) site-flanked *hph* cassette that contains a hygromycin resistance gene as selection marker in *S*. Enteritidis SJTUF12367, resulting in 26 TCS deletion mutants (see Fig. S1 in the supplemental material). The growth curve of these mutants was measured in LB. As shown in Fig. 1A, compared to the wild type (WT), the Δ*glnG*::*hph* mutant exhibited delayed growth (*P* < 0.05) during the logarithmic phase and the Δ*phoP*::*hph* mutant displayed a little lower culture density during the stationary phase, but there was no apparent change in bacterial growth rate in the rest of the TCS mutants. It was also found that there was no significant difference in the growth rates between the Δ*glnG* and WT strains when 0.4% glutamine was added to the culture: i.e., the glutamine could recover the delayed growth in the Δ*glnG* strain, indicating the importance of GlnGL in regulation of nitrogen source synthesis (Fig. 1B).

10.1128/msphere.00383-22.1 FIG S1 Confirmation of mutants by PCR. Lane 1, mutant’s genome amplification product, which contains an *hph* cassette; lane 2, WT genome amplification product. The format presenting the primer’s “gene name” followed by “_UJ-F/DJ-R” was used to check the changes in the amplicon sizes. Download FIG S1, TIF file, 0.8 MB. Copyright © 2022 Hu et al. 2022 Hu et al. https://creativecommons.org/licenses/by/4.0/ This content is distributed under the terms of the Creative Commons Attribution 4.0 International license.

10.1128/msphere.00383-22.9 TABLE S5 Primary primers used for mutagenesis and qRT-PCR. Download Table S5, DOCX file, 0.02 MB. Copyright © 2022 Hu et al. 2022 Hu et al. https://creativecommons.org/licenses/by/4.0/ This content is distributed under the terms of the Creative Commons Attribution 4.0 International license.

The MICs of the WT and 26 mutants against eight classes of antibiotics were determined, which covered the most common clinical and veterinary antibiotics. The results in Table 1 show that MIC values of 10 TCS mutants were significantly altered, while those of the other 16 TCS mutants were not apparently altered (data not shown). The Δ*arcA*::*hph* mutant showed 2- and 4-fold decreases in the MICs of tetracycline (TET) and azithromycin (AZI), whereas there were 2- and 4-fold increases in the MICs of streptomycin (STR) and gentamicin (GEN), respectively, compared to the WT. In addition, 2- and 16-fold decreases in the MICs of cefotaxime (CTX) and colistin (COL), respectively, for the Δ*basR*::*hph* mutant (*basR* is also known as *pmrB*), a 4-fold decrease in the MIC of GEN for the Δ*baeR*::*hph* mutant, a 4-fold increase in the MIC of GEN for the Δ*narP*::*hph* mutant, a 2-fold decrease in the MIC of CTX for the Δ*ompR*::*hph* mutant, a 2-fold decrease in the MIC of ceftriaxone (CRO) for the Δ*yehT*::*hph* mutant, and a 2-fold decrease in the MICs of ciprofloxacin (CIP) and ofloxacin (OFX) for the Δ*uvrY*::*hph* mutant were also observed.

**TABLE 1:** Susceptibility of *S.* Enteritidis and its derivatives to antibiotics

Antibiotic	Abbr	MIC (mg/L) of the *S*. Enteritidis (SJTUF12367) strain shown^*a*^													
		WT	Mutant												
			Δ*arcA*::*hph*	Δ*basR*::*hph*	Δ*yehT*::*hph*	Δ*baeR*::*hph*	Δ*narP*::*hph*	Δ*uvrY*::*hph*	Δ*ompR*::*hph*	Δ*glnG*	Δ*phoP*	Δ*cpxR*	Δ*glnG*::*glnG*	Δ*phoP*::*phoP*	Δ*cpxR*::*cpxR*
Piperacillin	PIP	512	512	512	512	512	512	512	512	128	512	512	512	512	512
Ceftazidime	CAZ	32	32	32	32	32	32	32	32	**8**	**4**	**8**	32	32	32
Ceftriaxone	CRO	256	256	256	**128**	256	256	256	256	**64**	**64**	**64**	256	256	256
Cefotaxime	CTX	256	256	**128**	256	256	256	256	**128**	**64**	**128**	**64**	256	256	256
Cefepime	FEP	32	32	32	32	32	32	32	32	**4**	**4**	**8**	32	32	32
Kanamycin	KAN	>512	>512	>512	>512	>512	>512	>512	>512	>512	>512	**128**	>512	>512	>512
Streptomycin	STR	256	512	256	256	256	256	256	256	**128**	256	**64**	256	256	256
Gentamicin	GEN	0.5	2	0.5	0.5	**0.12**	2	0.5	0.5	**0.12**	0.5	**0.12**	0.5	0.5	0.5
Nalidixic acid	NAL	256	256	256	256	256	256	256	256	256	**64**	**128**	256	256	256
Ofloxacin	OFX	0.5	0.5	0.5	0.5	0.5	0.5	**0.25**	0.5	0.5	**0.12**	**0.25**	0.5	0.5	0.5
Ciprofloxacin	CIP	0.12	0.12	0.12	0.12	0.12	0.12	**0.06**	0.12	**0.06**	**0.06**	**0.06**	0.12	0.12	0.12
Colistin	COL	8	8	**0.5**	8	8	8	8	8	8	**0.5**	8	8	8	8
Azithromycin	AZI	2	**0.5**	2	2	2	2	2	2	**0.25**	2	2	2	2	2
Tetracycline	TET	128	**64**	128	128	128	128	128	128	**32**	128	128	128	128	128
Chloramphenicol	CHL	256	256	256	256	256	256	256	256	256	256	256	256	256	256
Trimethoprim-sulfonamides	SXT	>32/608	>32/608	>32/608	>32/608	>32/608	>32/608	>32/608	>32/608	**16/304**	>32/608	>32/608	>32/608	>32/608	>32/608
Fosfomycin	FOS	>512	>512	>512	>512	>512	>512	>512	>512	>512	>512	>512	>512	>512	>512

a The antibiotic susceptibilities of the Δg*lnG*::*hph*, Δ*phoP*::*hph*, and Δ*cpxR*::*hph* mutants were consistent with those of the Δ*glnG*, Δ*phoP*, and Δ*cpxR* mutants, respectively. The Δ*glnG*::*glnG*, Δ*phoP*::*phoP*, and Δ*cpxR*::*cpxR* strains are the complementary strains of the Δ*glnG*, Δ*phoP*, and Δ*cpxR*, mutants, respectively. Values above or below the WT MICs are underlined or in boldface, respectively. Abbr, abbreviation.

It was found that deletion of the gene *cpxR*, *phoP*, or *glnG* influenced the susceptibility of *S*. Enteritidis to at least three classes of antibiotics. Compared to the WT, there was a 16-fold decrease in the MIC of colistin in the Δ*phoP*::*hph* mutant and there was a 2- to 4-fold decrease in the MIC of aminoglycosides (STR, GEN, and kanamycin [KAN]) in the Δ*cpxR*::*hph* mutant. Both Δ*phoP*::*hph* and Δ*cpxR*::*hph* strains showed 2- to 8-fold decreases in the MICs of cephalosporins (CAZ, CRO, CTX, and cefepime [FEP]) and quinolones (NAL, CIP, and OFX). Likewise, there was a 2- to 8-fold reduction in resistance to antibiotics, including piperacillin (PIP), CAZ, CRO, CTX, FEP, STR, GEN, AZI, TET, and sulfamethoxazole-trimethoprim (SXT) in the Δ*glnG*::*hph* mutant. These results were also consistent with the resistant phenotype in the *hph* cassette-free Δ*cpxR*, Δ*glnG*, and Δ*phoP* mutant strains (Fig. 1C), respectively, while the complementary strains Δ*cpxR*::*cpxR*, Δ*glnG*::*glnG*, and Δ*phoP*::*phoP* complementary strains (Fig. S2) showed no obvious difference in MIC values compared with the WT (Table 1).

10.1128/msphere.00383-22.2 FIG S2 Schematics of *phoP* complementation. The entire coding region of *phoP* along with its ribosome-binding site and restriction enzyme sites (EcoRI and XbaI) were amplified and cloned into the pBAD33 plasmid, which was transformed into Δ*phoP* to construct the strain with *phoP* complementation (Δ*phoP*::*phoP*). Download FIG S2, TIF file, 0.3 MB. Copyright © 2022 Hu et al. 2022 Hu et al. https://creativecommons.org/licenses/by/4.0/ This content is distributed under the terms of the Creative Commons Attribution 4.0 International license.

### CpxAR, PhoPQ, and GlnGL played an important role in developing MDR in different S. Enteritidis isolates.

To determine the roles of CpxAR, PhoPQ, and GlnGL in regulation of drug resistance of various *S*. Enteritidis isolates, six clinical MDR isolates (SE1 to -6), which exhibited resistance to cephalosporins and nalidixic acid and/or ACSSuT antimicrobial resistance profiles (Table S1), were used to construct deletion mutants with single gene deletions of *cpxR*, *phoP*, and *glnG*, respectively. The resulting deletion mutants were verified by PCR and sequencing (Fig. S3). The MICs for SE1 to -6 and their mutants against quinolones, cephalosporins, aminoglycosides, colistin, and tetracycline were then determined. As shown in Table 2, these wild-type (WT) isolates exhibited resistance to CAZ, CRO, CTX, FEP, and NAL. Among the 6 *cpxR* deletion mutants, 4 showed 2- to 8-fold decreases in the MICs of CAZ, CRO, OFX, NAL, STR, KAN, and GEN compared with their parental strains. In addition, all 6 of the *phoP* deletion mutants showed 2- to 16-fold decreases in the MICs of CAZ, CRO, FEP, CIP, OFX, NAL, and COL. Most of the Δ*glnG*::*hph* mutants showed 2- to 8-fold decreases in the MICs of CAZ, CRO, FEP, CIP, and TET, and a few mutants exhibited higher susceptibility to OFX, NAL, STR, or KAN than their parental strains. The changes in antibiotic sensitivity of these mutants were consistent with those caused by *cpxR*, *phoP*, or *glnG* deletion in *S*. Enteritidis SJTUF12367. Altogether, CpxAR, PhoPQ, and GlnGL all modulated resistance of *S*. Enteritidis to quinolones and cephalosporins, in which CpxAR also facilitated resistance to aminoglycosides, PhoPQ conferred resistance to colistin, and GlnGL mediated resistance to aminoglycosides and tetracycline. These results demonstrated that CpxAR, PhoPQ, and GlnGL contributed to the development of multiple-drug resistance in *S.* Enteritidis MDR isolates.

**TABLE 2:** Susceptibility of *S*. Enteritidis isolates to antibiotics

Strain^*a*^	MIC (mg/L) of^*b*^:											
	CAZ	CRO	CTX	FEP	CIP	OFX	NAL	STR	KAN	GEN	COL	TET
SE1 WT	64	256	256	16	0.125	0.5	512	512	>512	0.5	8	64
Mutant												
Δ*cpxR::hph*	**32**	**128**	**128**	**8**	0.125	0.5	**256**	**64**	**128**	0.5	8	64
Δ*phoP*::*hph*	**16**	**64**	**128**	**8**	**0.0312**	**0.125**	**256**	**256**	**512**	0.5	**1**	64
Δ*glnG*::*hph*	**16**	256	256	**8**	0.125	0.5	512	**256**	>512	0.5	8	**16**
SE2 WT	64	256	256	16	0.125	0.25	256	256	512	0.5	4	32
Mutant												
Δ*cpxR*::*hph*	**32**	**128**	256	**8**	**0.0625**	**0.125**	**128**	**64**	**128**	**0.25**	4	32
Δ*phoP*::*hph*	**16**	**128**	256	**8**	**0.0312**	**0.0625**	**64**	**256**	512	0.5	**0.5**	32
Δ*glnG*::*hph*	**16**	**128**	256	**8**	**0.0625**	**0.125**	**128**	**128**	512	**0.25**	4	32
SE3 WT	64	256	128	16	0.125	0.125	256	16	512	0.5	2	4
Mutant												
Δ*cpxR*::*hph*	**32**	**128**	**128**	16	**0.0312**	**0.0625**	**128**	**4**	**128**	**0.125**	2	4
Δ*phoP*::*hph*	**32**	**128**	**64**	**8**	**0.0312**	**0.0312**	**128**	16	512	0.5	**0.25**	4
Δ*glnG*::*hph*	**16**	**64**	**64**	**8**	0.125	0.125	256	16	512	0.5	2	**2**
SE4 WT	64	256	128	16	0.125	0.25	256	16	16	0.5	4	8
Mutant												
Δ*cpxR*::*hph*	**32**	**128**	**32**	16	0.125	**0.0625**	**128**	**4**	**2**	**0.25**	4	8
Δ*phoP*::*hph*	**32**	**128**	**64**	**8**	**0.0312**	**0.0625**	**128**	16	**8**	0.5	**0.25**	8
Δ*glnG*::*hph*	**16**	**128**	128	**8**	**0.0312**	**0.125**	256	16	16	0.5	4	**2**
SE5 WT	64	256	256	16	0.25	0.5	512	512	>512	0.5	8	32
Mutant												
Δ*cpxR*::*hph*	**32**	**128**	**128**	16	**0.125**	**0.25**	512	**128**	**128**	**0.25**	**4**	32
Δ*phoP*::*hph*	**16**	**32**	**128**	**8**	**0.125**	**0.25**	**128**	**256**	**512**	0.5	**0.5**	32
Δ*glnG*::*hph*	**16**	**128**	256	16	**0.125**	0.5	512	**256**	**512**	0.5	8	**16**
SE6 WT	64	256	256	32	0.125	0.25	256	256	512	1	8	32
Mutant												
Δ*cpxR*::*hph*	**32**	**128**	**128**	32	0.125	**0.125**	**128**	**128**	**256**	**0.25**	8	32
Δ*phoP*::*hph*	**16**	**128**	**128**	**16**	**0.0625**	**0.0625**	**128**	256	512	1	**0.5**	32
Δ*glnG*::*hph*	**16**	**128**	256	**16**	0.125	**0.125**	**128**	**128**	512	1	8	**16**

a SE1 to -6 are *S.* Enteritidis isolates SJTUF14364, SJTUF14365, SJTUF14409, SJTUF14745, SJTUF14749, and SJTUF14751, respectively.

b CAZ, ceftazidime; CRO, ceftriaxone; CTX, cefotaxime; FEP, cefepime; CIP, ciprofloxacin; OFX, ofloxacin; NAL, nalidixic acid; STR, streptomycin; KAN, kanamycin; GEN, gentamicin; COL, colistin; TET, tetracycline. Values below the parent strain’s MIC are marked in boldface.

10.1128/msphere.00383-22.3 FIG S3 Construction and confirmation of the *cpxR*, *phoP*, and *glnG* mutants. (A) Amplification of the upstream flank (UF) and downstream flank (DF) of *cpxR*, *phoP*, and *glnG*; (B) amplification of the *hph* cassette; (C to E) splicing of homologous recombination fragments (UF, *hph* cassette plus DF) of *cpxR* (C), *phoP* (D), and *glnG* (E) by overlap-extension PCR. HRF, homologous recombination fragment. (F to K) Confirmation of mutants by PCR. The format presenting the primer’s “gene name” followed by “_In-F/R” was used to check the product sizes of *cpxR* (F), *phoP* (G), and *glnG* (H). The format presenting the primer’s “gene name” followed by “_UJ-F/DJ-R” was used to check the product size of *cpxR* plus flanking sequences (I), *phoP* plus flanking sequences (J), and *glnG* plus flanking sequences (K). The primers used and expected amplicon sizes are given in Table S5. Lanes: con, wild-type strain of SJTUF12367; 1 to 6, mutant derivates from SJTUF14364, SJTUF14365, SJTUF14409, SJTUF14745, SJTUF14749, and SJTUF14751 (SE1 to -6), respectively. Download FIG S3, TIF file, 0.5 MB. Copyright © 2022 Hu et al. 2022 Hu et al. https://creativecommons.org/licenses/by/4.0/ This content is distributed under the terms of the Creative Commons Attribution 4.0 International license.

10.1128/msphere.00383-22.5 TABLE S1 Primary strains and plasmids used in this study. Download Table S1, DOCX file, 0.01 MB. Copyright © 2022 Hu et al. 2022 Hu et al. https://creativecommons.org/licenses/by/4.0/ This content is distributed under the terms of the Creative Commons Attribution 4.0 International license.

### CpxAR and PhoPQ directly interacted with ARGs.

**FIG 2:**
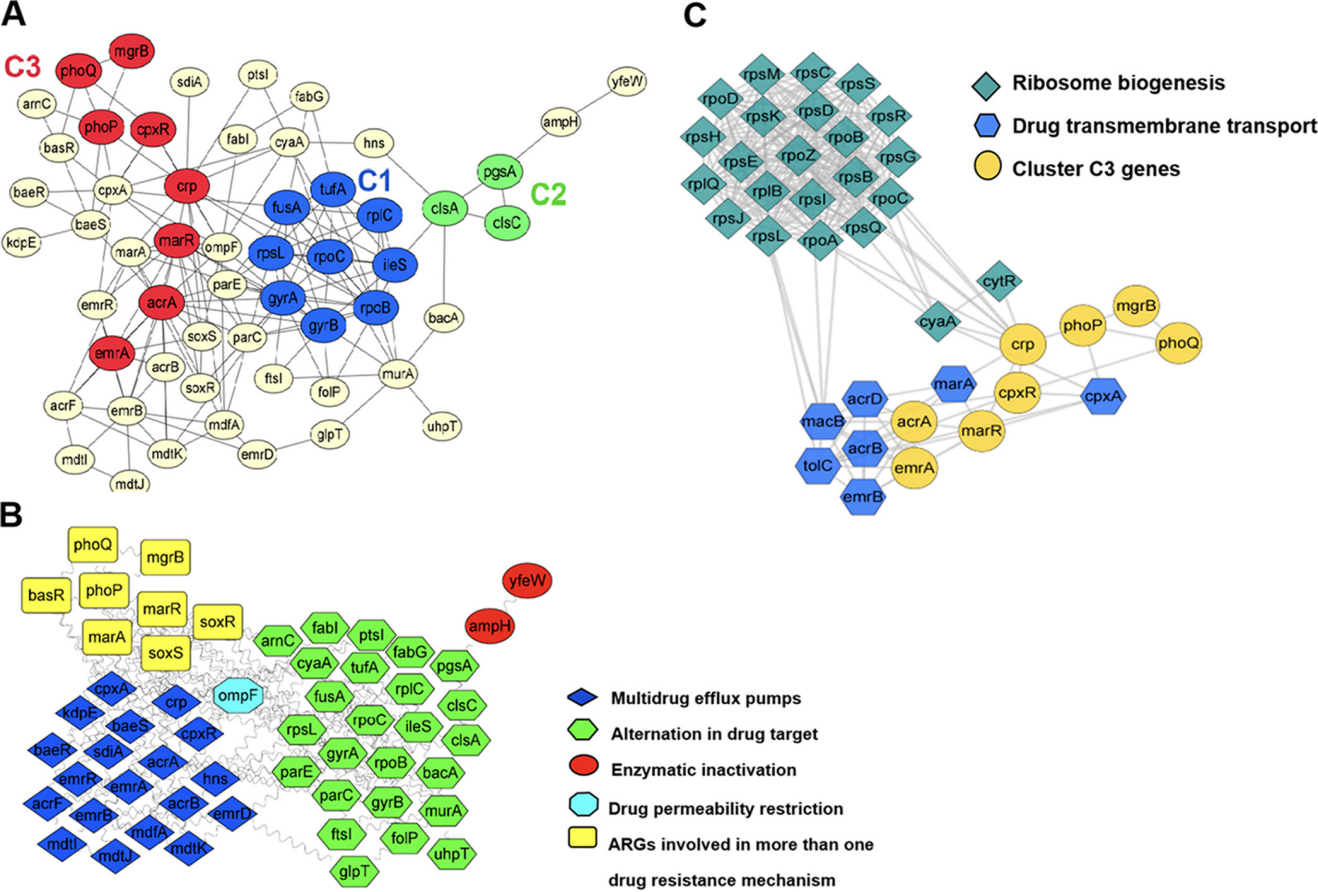
Analysis of interaction of CpxAR and PhoPQ with antimicrobial resistance genes. (A) Overall graphical representation of interaction network on 55 ARGs of S. Enteritidis. The interaction between two genes (nodes) is represented in colored lines (edges), and it represents evidence derived from various sources (high-throughput experimental data, mining of databases, literature, and analyses of coexpressed genes). Three gene clusters are marked as C1 to C3. Each cluster has been given a different color, and the genes not included in clusters are shown in pale yellow. (B) Classification of drug resistance mechanisms of ARGs. ARGs involved in each mechanism are represented in different colors based on the drug resistance mechanism. (C) Extended network of C3 genes. The interaction network of C3 ARGs was extending to 38 node genes and 264 interaction edges, and the average interaction score of each node gene was 13.9. C3 genes are shown as ellipses, whereas functional partners are shown as diamonds and hexagons according to their gene function. The nodes were given different colors based on their function category.

To characterize the interactions between important TCSs (*cpxAR*, *phoPQ*, and *glnGL*) and ARGs, the ARGs of the SJTUF12367 genome were collected from the 3 databases PATRIC, CARD, and NDARO and analyzed. A total of 127 ARGs (33 in PATRIC, 39 in CARD, and 55 in NDARO) were obtained, and 78 nonredundant ARGs were screened out. Finally, 55 ARGs (including *cpxAR* and *phoPQ*) showed interactions in the STRING database along with 163 functional interactions, and three densely interconnected clusters (designated C1 to -3) were obtained via Cytoscape (Fig. 2A). However, string analysis showed that *glnGL* had no interaction links with ARGs.

These ARGs were responsible for various resistance mechanisms, including multidrug efflux pumps (19 genes), alteration in drug targets (25 genes), enzymatic inactivation/degradation (2 genes), drug permeability restriction (1 gene), and MDR mechanisms (8 genes) (Fig. 2B). Additionally, functional enrichment analysis showed that C3 genes (*phoP*, *phoQ*, *crp*, *marR*, *acrA*, *mgrB*, *emrA*, and *cpxR*) were enriched in the pathway of cationic antimicrobial peptide resistance (eco01503), response to stimulus (GO:0050896), drug transmembrane transporter activity (GO:0015238), and DNA binding (GO:0003677), most of which were involved in the MDR mechanisms (data not shown).

To explore the ARGs’ correlation with *cpxAR* and *phoPQ*, the extended network with C3 genes was analyzed. The functional partners of C3 genes were classified into class I and II based on their functions via STRING clusters (Fig. 2C and Table S2). The genes in class I (CL:46) consisted of *rpo*, *rps*, and *rpl* operons, which encoded RNA polymerase (RNAP) and the 30S and 50S ribosomal subunits, respectively, and were responsible for ribosome assembly. Another observation in class II (CL:3574) showed a high number of interconnections among efflux pump genes (*acrAB*, *acrD*, *macB*, and *emrAB*), global regulator genes (*marA* and *crp*), and the *marRAB* operon repressor gene (*marR*), which were enriched in the pathway of response to antibiotic (GO:0046677) and transmembrane transporter complex (GO:1902495). In Salmonella, the tripartite complex AcrAB-TolC extruded multiple antibiotics, such as β-lactams, quinolones, and tetracyclines, and gave rise to MDR. Other efflux pumps (EmrAB, AcrD, and MacB) also expelled specific drugs and would in a way compensate for the loss of function of the inactivated AcrAB pump (30). The global transcriptional regulators, not limited to MarA, upregulated *acrAB* expression and played an important role in compensatory regulation at a global level (30). Therefore, the class II genes were responsible for antibiotic transmembrane export by enhancing efflux pump activity.

10.1128/msphere.00383-22.6 TABLE S2 Functional enrichment of C3 extended network genes. Download Table S2, DOCX file, 0.02 MB. Copyright © 2022 Hu et al. 2022 Hu et al. https://creativecommons.org/licenses/by/4.0/ This content is distributed under the terms of the Creative Commons Attribution 4.0 International license.

The interactions of *cpxAR* and *phoPQ* with global regulatory factors were presented in the C3 extended networks, which included the interconnections among *cpxAR*, *marA*, *tolC*, and *crp* and the interactions between *phoPQ* and *crp*. These association cues were somehow coordinated through a coupling of CpxAR or PhoPQ signaling. It has been reported that CpxAR responded to envelope damage, followed by modulation of transcription of *marA*, activation of *acrAB*-*tolC* efflux, and alteration of expression of some outer membrane (OM) porin genes (*ompC* and *ompF*) in E. coli (31). The cAMP receptor protein (CRP) encoded by *crp* could recognize the RNA polymerase (RNAP)-binding site and modulate expression of hundreds of genes through interfacial interactions with RNAP (32); it was found that loss of *crp* resulted in an increase in resistance to fluoroquinolones in *S.* Typhimurium via reduced expression of OM porin genes (*ompA*, *ompC*, and *ompF*) and increased expression of an efflux pump gene (*acrB*) (33). Therefore, the interaction results proposed that CpxAR and PhoPQ of *S*. Enteritidis might affect the function of global regulators to enhance the fine-tuning of efflux pump and OM porin genes.

### Deletion of *phoP*/*cpxR* resulted in an increase in cell membrane permeability.

The relative mRNA expression of a series of ARGs among WT and Δ*phoP*/Δ*cpxR* cells was determined via quantitative reverse transcription-PCR (qRT-PCR). In this work, ceftazidime (CAZ) and nalidixic acid (NAL) were used as the representatives of quinolones and cephalosporins, respectively. The subinhibitory concentrations of 2 mg/L CAZ (1/16 MIC) and 32 mg/L (1/8 MIC) NAL were selected because all strains could grow at these concentrations, and the population density of Δ*cpxR*/Δ*phoP* mutants was able to reach a WT level (Fig. S4).

10.1128/msphere.00383-22.4 FIG S4 Growth curves of the WT and Δ*phoP* and Δ*cpxR* mutants at subinhibitory concentrations of antibiotics. (A) Subinhibitory concentrations of nalidixic acid (NAL), ceftazidime (CAZ), or chloramphenicol (CHL) differentially affect growth of Δ*phoP*, Δ*cpxR*, and WT cells. Cell densities were measured as the OD_600_ at 6 h of growth with exposure to antibiotic at the indicated concentrations. The WT and mutants were equally susceptible to subinhibitory concentrations of CHL. Compared to WT cells, incubation of the Δ*phoP* and Δ*cpxR* mutants with NAL at the concentration exceeding 1/8 MIC or with CAZ at the concentration exceeding 1/16 MIC resulted in growth inhibition. (B) Cell densities were measured for 24 h with exposure to antibiotic at the indicated concentrations. Growth curves present the average of 3 biological replicates. In the absence of antibiotics, the Δ*phoP* and Δ*cpxR* mutants have similar growth curve characteristics to WT strains. Download FIG S4, TIF file, 0.6 MB. Copyright © 2022 Hu et al. 2022 Hu et al. https://creativecommons.org/licenses/by/4.0/ This content is distributed under the terms of the Creative Commons Attribution 4.0 International license.

**FIG 3:**
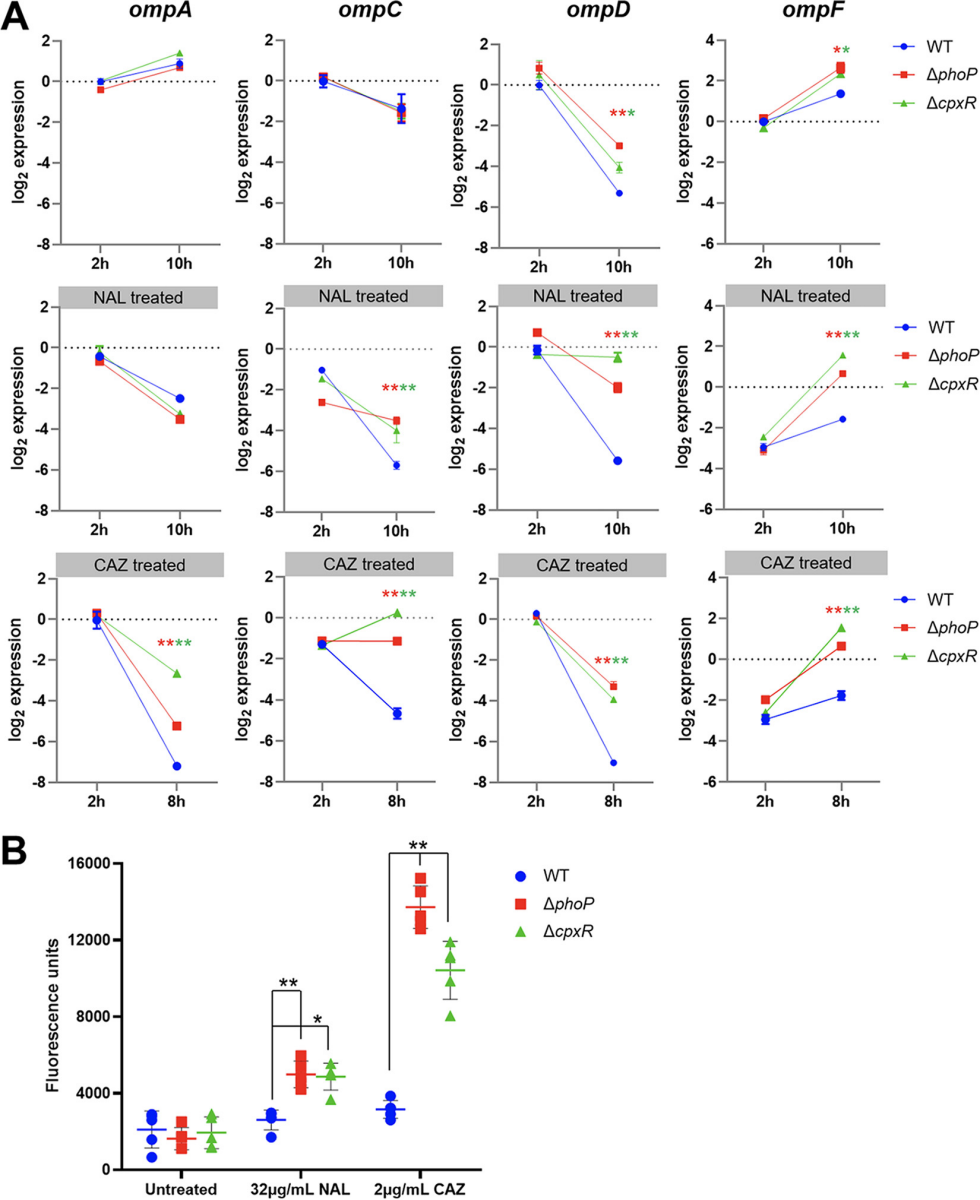
CpxAR and PhoPQ modulate membrane permeability. (A) Expression analysis of outer membrane porin genes in Δ*phoP*, Δ*cpxR*, and WT cells. Shown are results from qRT-PCR analysis of the mRNA expression levels of the *ompA*, *ompC*, *ompD*, and *ompF* genes in cells untreated (top), treated with 32 mg/L of NAL (middle), and treated with 2 mg/L of CAZ (bottom). All points were expressed relative to the untreated WT at 2 h, represented by the dotted gray line. Significance analysis was determined by the Holm-Sidak method. Error bars represent 95% confidence intervals. (B) NPN uptake assay. Error bars indicate the standard deviation of the results from 5 technical replicates. *, *P* < 0.05; **, *P* < 0.01.

The porins OmpA, OmpC, OmpD, and OmpF were abundant in bacterial outer membrane, among which OmpC and OmpF were linked directly to ciprofloxacin resistance (34), while OmpA was a large porin that allowed for slow diffusion of molecules at a rate 50 times lower than that of OmpF (35). As shown in Fig. 3A, the transcriptional levels of the *ompA*, *ompC*, *ompD*, and *ompF* genes were measured at the bacterial exponential growth phase (2 h) and the early stationary phase (8 h or 10 h) under the unstressed condition. Compared to WT cells, the transcriptional levels of *ompC* and *ompA* genes were not significantly different among all mutants. However, deletion of *cpxR* or *phoP* resulted in higher expression levels of *ompD* and *ompF* genes than the WT (*P* < 0.05) after 10 h, which indicated that the homeostasis of bacterial envelope was disturbed.

To test the hypothesis that the Δ*cpxR/*Δ*phoP* mutants were more permeable to small-molecule antibiotics than the WT, we treated bacterial cells with subinhibitory concentrations of ceftazidime (2 mg/L) or nalidixic acid (32 mg/L). As expected, the differences in the expression of porins between the WT and mutants were enlarged after adding antibiotics (Fig. 3A). The expression levels of the *ompC*, *ompD*, and *ompF* genes were significantly (*P* < 0.01) higher in Δ*cpxR/*Δ*phoP* mutants than in the WT at the early stationary phase. The expression level of the *ompA* gene did not apparently differ among all strains under NAL pressure, but expression was significantly (*P* < 0.001) higher in mutants than the WT under CAZ stress for 8 h.

Permeabilization activity against membranes of *S*. Enteritidis wild-type and Δ*cpxR*/Δ*phoP* mutants was determined using 1-*N*-phenylnaphthylamine (NPN) uptake assays (36). As shown in Fig. 3B, the fluorescence intensity of NPN was not significantly changed among WT cells not under stress or with CAZ and NAL added, indicating that subinhibitory concentrations of antibiotics did not destroy the OM permeability of WT. In addition, the fluorescence intensities of WT, Δ*phoP*, and Δ*cpxR* cells were similar without antibiotic treatment. However, compared to WT cells, the NPN fluorescence intensity was significantly increased in Δ*cpxR* and Δ*phoP* cells after adding antibiotics and was especially markedly increased in the Δ*cpxR* and Δ*phoP* cells under the ceftazidime condition (*P* < 0.01). The difference between NAL- and CAZ-treated cells could arise from severe membrane damage by β-lactam-mediated perturbation of peptidoglycan biosynthesis, which led to destabilization or lysis of the cellular envelope. Overall, CpxAR and PhoPQ could maintain and modify bacterial membrane permeability by regulating expression of OM porins and result in an increase in the resistance of *S*. Enteritidis to quinolones and cephalosporins.

### CpxAR and PhoPQ strengthened the specific efflux pump activity.

**FIG 4:**
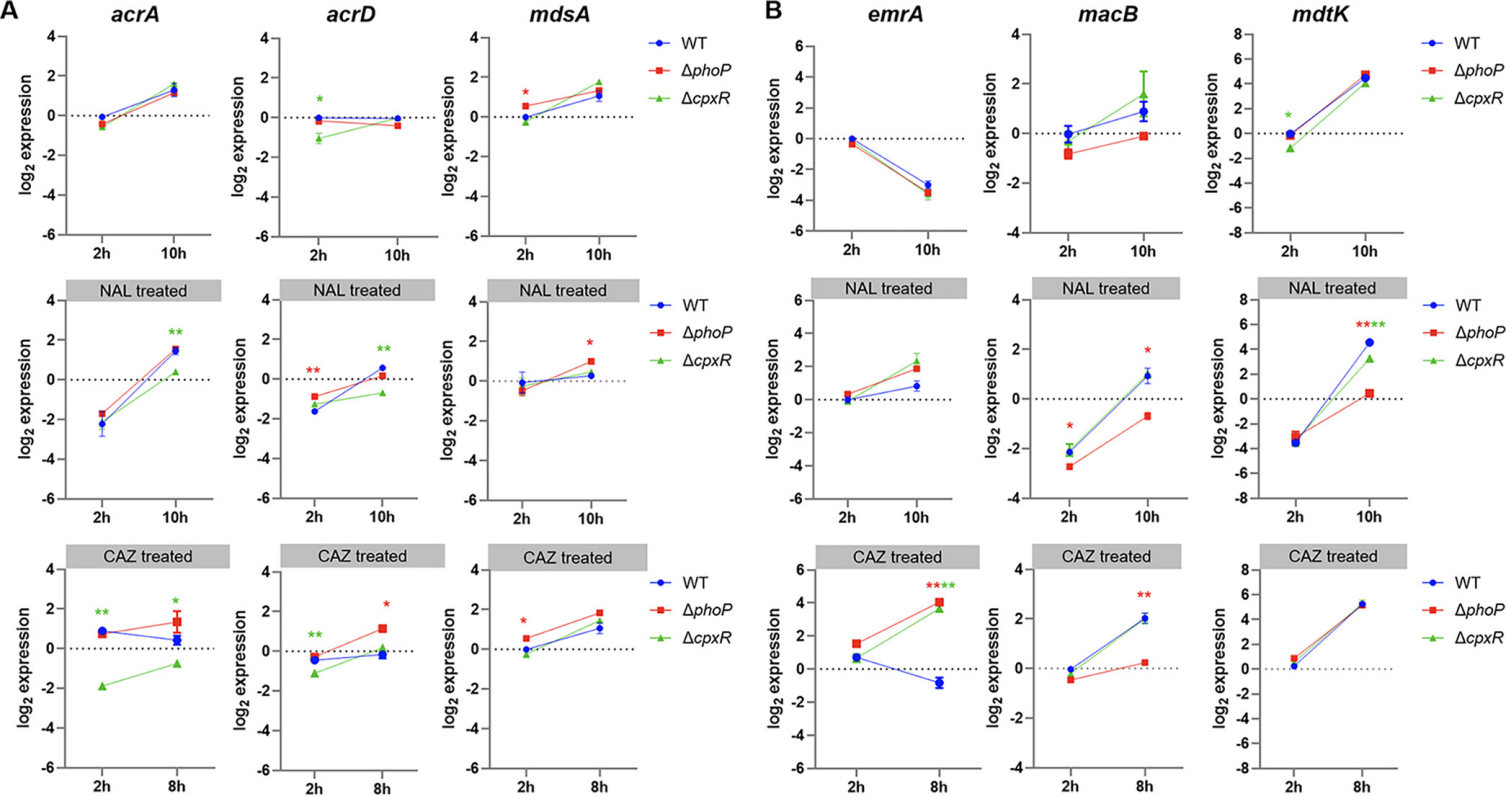
Transcriptional levels of efflux pump genes. Shown are the mRNA expression levels of RND efflux pump genes (A) and other efflux pump genes (B) in cells untreated (top), treated with 32 mg/L of NAL (middle), and treated with 2 mg/L of CAZ (bottom). All points are expressed relative to the untreated WT at 2 h, represented by the dotted gray line. *, *P* < 0.05; **, *P* < 0.01; ***, *P* < 0.001.

To characterize the role of PhoPQ and CpxAR in the function of efflux pumps, qRT-PCR was carried out with or without antibiotic to test the transcription characteristics of 4 families of efflux pump genes, including the resistance-nodulation-cell division (RND) family pumps (AcrA, AcrD, and MdsA), the major facilitator superfamily (MFS) pump (EmrA), the multidrug and toxic compound extrusion (MATE) family pump (MdtK), and the ATP-binding cassette (ABC) family pump (MacB). After an incubation for 10 h with nalidixic acid, Δ*phoP* mutant cells were shown to have significantly reduced expression levels of the *macB* and *mdtK* genes, while the Δ*cpxR* mutant had lower expression levels of the *acrA* and *acrD* genes (*P* < 0.001) than the WT (Fig. 4A and B). After ceftazidime treatment for 8 h, there was no significant alteration in the expression levels of *macB* and *mdtK* genes in the Δ*cpxR* mutant compared to the WT, whereas the expression levels of *acrA* in the Δ*cpxR* mutant and *macB* in the Δ*phoP* mutant were significantly decreased (*P* < 0.01). In addition, at the early stationary stage of cultivation after antibiotic treatment, extremely increased expression levels of the *emrA* gene in Δ*cpxR* and Δ*phoP* cells were achieved compared to those of WT cells. These results demonstrated that *phoP* and *cpxR* promoted bacterial resistance to NAL and CAZ through regulation of the expression of efflux pumps. In this case, PhoP played a positively regulatory role in MacB and MdtK pumps, while CpxR had an obvious effect on activation of RND pumps; in contrast, the partial abnormalities of aforementioned efflux pumps might be neutralized or compensated via overexpression of other pumps (e.g., EmrA) under antibiotic pressure at subinhibitory concentrations.

### CpxAR and PhoPQ modulated the expression of specific global regulatory factors.

**FIG 5:**
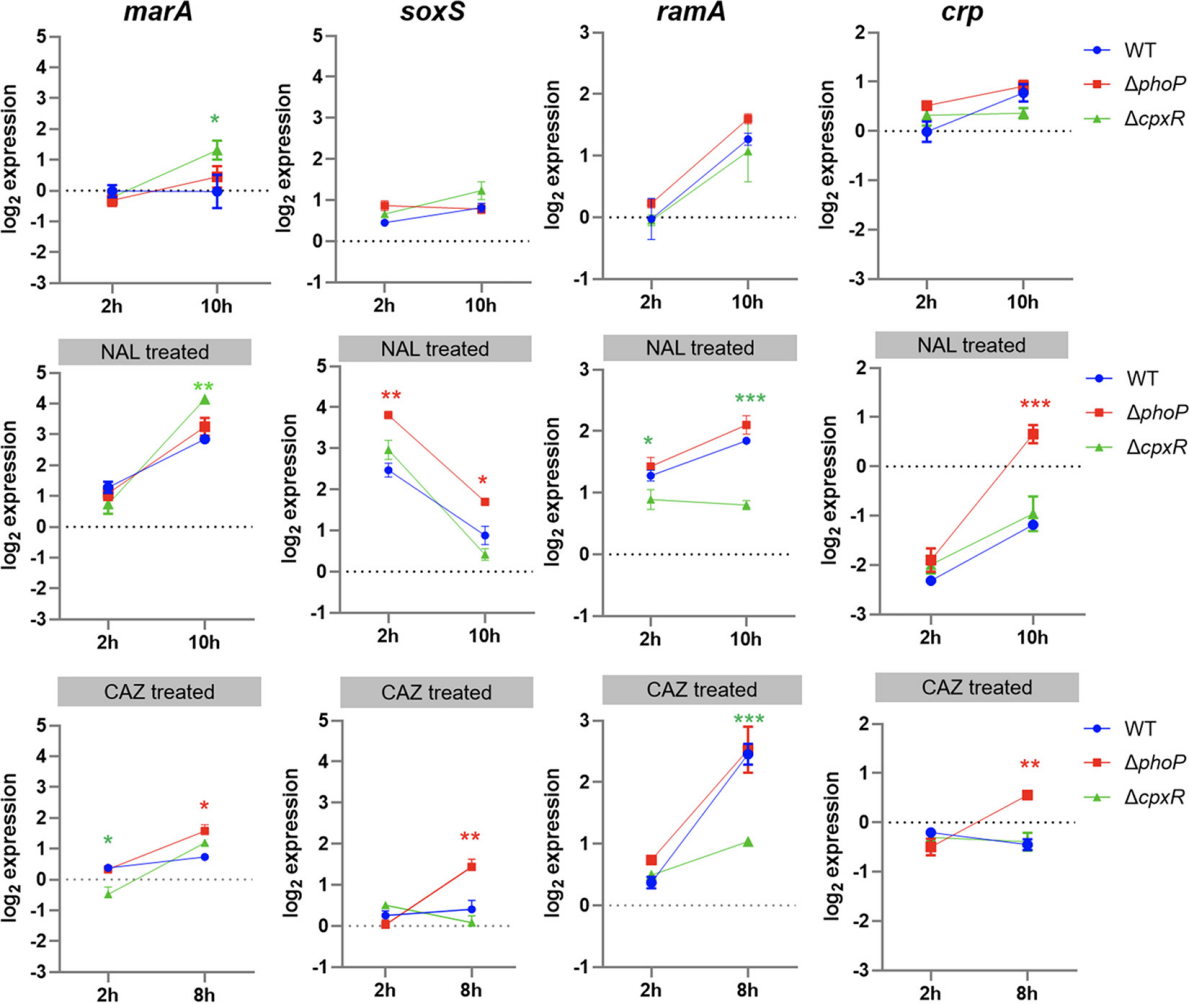
Transcriptional levels of global regulators. Shown are the expression levels of the *marA*, *soxS*, *ramA*, and *crp* genes in cells untreated (top), treated with 32 mg/L of NAL (middle), and treated with 2 mg/L of CAZ (bottom). All points are expressed relative to the untreated WT at 2 h, represented by the dotted gray line. *, *P* < 0.05; **, *P* < 0.01; ***, *P* < 0.001.

As presented in the C3 extended network (Fig. 2C), the global regulatory factors were key nodes in CpxAR- and PhoPQ-mediated regulation of efflux pump genes. qRT-PCR results showed that there were 2- to 4-fold increases in the expression levels of the *marA*, *soxS*, and *ramA* genes and a 4-fold decrease in the expression level of *crp* gene in NAL-treated cells, whereas no significant difference was observed in CAZ-treated cells compared to untreated WT cells at 2 h (Fig. 5). When cells were treated at the early stationary stage, the expression level of *ramA* was significantly lower in the Δ*cpxR* mutant, but in the Δ*phoP* mutant, *ramA* expression was similar to or higher than that in the WT cells. In addition, the expression level of the *crp* gene in Δ*phoP* mutant was significantly (*P* < 0.001) increased, which had not been identified in previous studies under antibiotic conditions, indicating the negative regulation of PhoP on *crp* expression. Furthermore, the expression level of *marA* gene was increased and higher in all mutant cells than in the WT, indicating that MarA possessed the compensatory function to overcome subinhibitory concentrations of antibiotics. Therefore, the lack of CpxR not PhoP would impair the global regulator of RamA, thereby indirectly and positively regulating the function of RND efflux pump; correspondingly, PhoP acts in a *crp*-dependent manner.

### There existed a compensatory function between PhoP and CpxR.

**FIG 6:**
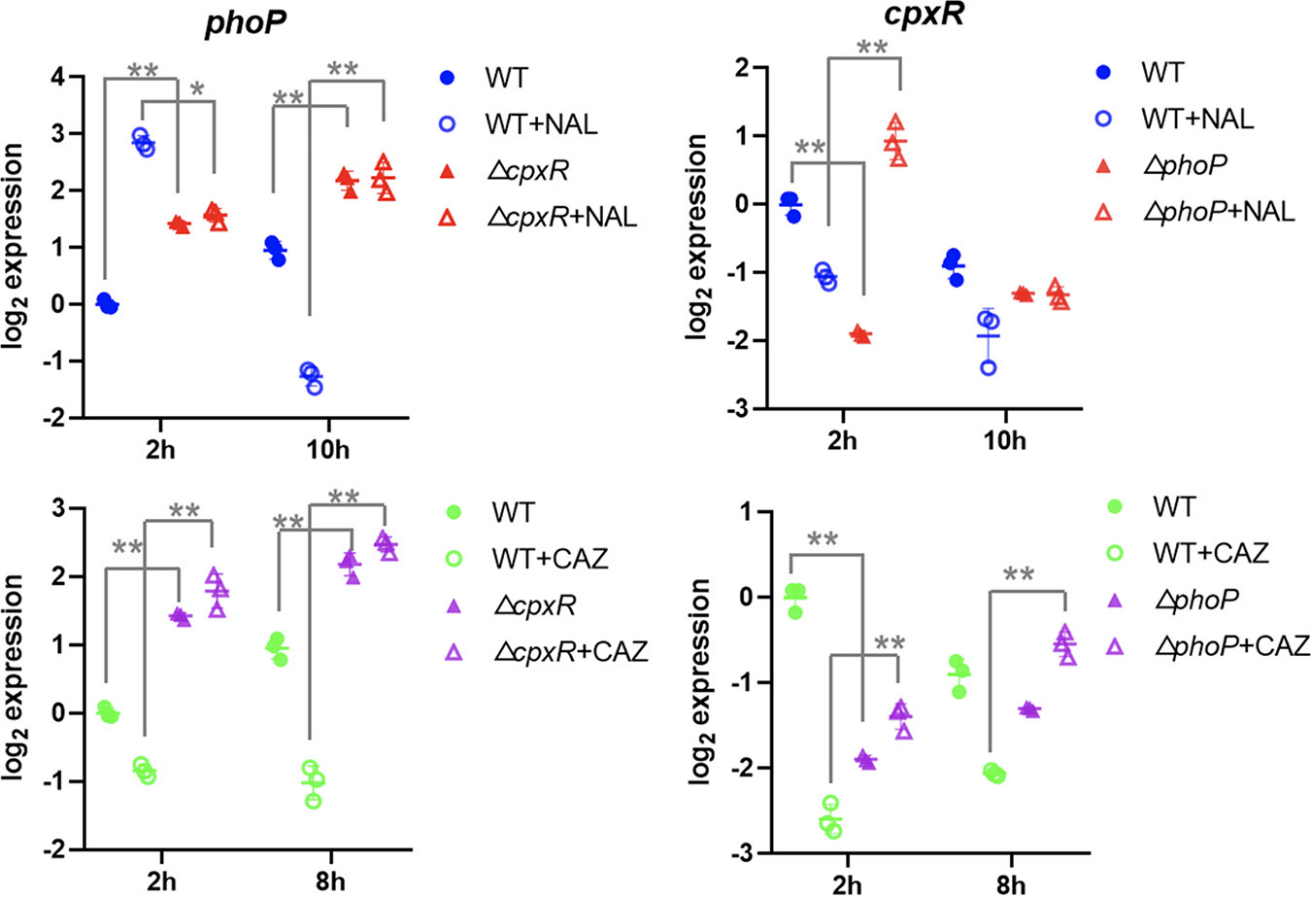
Transcriptional levels of *phoP* and *cpxR* genes. Shown are the expression levels of the *phoP* and *cpxR* genes in cells untreated, treated with 32 mg/L of NAL, and treated with 2 mg/L of CAZ. All points are expressed relative to untreated WT at 2 h. *, *P* < 0.05; **, *P* < 0.01.

The expression levels of the *phoP* and *cpxR* genes in the WT and mutants were determined. Although the expression level of *phoP* showed no significant difference among Δ*cpxR*, NAL-treated Δ*cpxR* (Δ*cpxR*+NAL) cells, and CAZ-treated Δ*cpxR* (Δ*cpxR+*CAZ) cells, a comparatively higher expression level of *phoP* was found in Δ*cpxR* cells than in WT cells (*P* < 0.05) (Fig. 6). Similarly, the expression level of *cpxR* in Δ*phoP* cells was significantly higher than those in WT cells. In addition, significantly higher expression levels of *cpxR* in Δ*phoP*+NAL or Δ*phoP*+CAZ cells than untreated Δ*phoP* cells were also observed (except for NAL treatment at 10 h). These data indicated that there was a compensation mechanism between *phoP* and *cpxR* functions, which might partially account for the antibiotic pressure fitness of *S*. Enteritidis.

## DISCUSSION

In the present study, a large-scale mutational analysis was conducted on MDR *S*. Enteritidis to explore the role of TCSs in the development of antibiotic resistance. Our results revealed that 38.46% of the TCS pairs were involved in antibiotic resistance of *S*. Enteritidis. Among these TCSs, BarA/UvrY, NarQP, and ArcBA, previously known as regulators for metabolic control (37–39), were found to facilitate resistance development. Additionally, the absence of *yehT* and *basR* resulted in an increase in susceptibility of *S*. Enteritidis to ceftriaxone and colistin, respectively, in agreement with published studies where overexpression of *yehT* caused a 2-fold increase in MIC of β-lactams and mutation in PmrAB resulted in a decrease in resistance to colistin (19, 40). However, in the present study, the mutants with deletion of *baeR* and *ompR* exhibited increased susceptibility to gentamicin and cefotaxime, respectively, whereas overexpression of *baeR* was previously reported to lead to increased resistance to ceftiofur and cefotaxime, and alteration of EnvZ/OmpR contributed to ertapenem and nalidixic acid resistance in E. coli (41–43). Thus, there were differences in the effects of TCS orthologs on bacterial resistance.

The TCSs CpxAR, PhoPQ, and GlnGL were shown to play important roles in MDR of *S*. Enteritidis SJTUF12367, which was observed in multiple *S*. Enteritidis isolates, indicating the universal contribution of these TCSs to the MDR phenotype of *S*. Enteritidis serovars. It was previously reported that the absence of *phoPQ* led to attenuated expression of *mltD1* and *slt* and increased β-lactam influxes in Stenotrophomonas maltophilia (44), and inactivation of CpxR resulted in higher susceptibility to aminoglycosides and β-lactams of *S*. Typhimurium (18). In our findings, *phoP* and *cpxR* mutants additionally exhibited higher susceptibility to nalidixic acid, ciprofloxacin, and ofloxacin with a drastic decrease (up to 8-fold) in MIC, suggesting a more extensive influence of these TCSs on antibiotic resistance. GlnGL (also called NtrBC) was a key regulator in response to nitrogen source and nitrogen availability via activation of expression of *glnA*, which encoded a glutamine synthetase (GS) (45). Recently, a few clues in susceptible strains showed that the absence of *ntrBC* in E. coli and *glnA* in Salmonella enterica serovar Typhi could increase their susceptibility to aminoglycosides and ciprofloxacin, respectively (46, 47). In this study, a phenotype in *S*. Enteritidis *glnG* mutants of relative susceptibility to quinolones and aminoglycosides was observed, and the absence of *glnG* also led to an increase in susceptibility to cephalosporins and tetracycline, suggesting a greater influence of *glnG* on MDR *S*. Enteritidis than on susceptible strains. Overall, these TCSs were crucial for development of multidrug resistance in *S*. Enteritidis.

Gene network analysis is one of the reliable approaches that provide new insights into biological information of antimicrobial resistance mechanisms. In this work, the ARGs were placed into three clusters. The genes in cluster C1 (e.g., *gyrA*, *rpoB*, and *rplC*) were responsible for alteration in drug targets (48–52); however, there was only one single-base mutation in GyrA, D87G, found in the SJTUF12367 genome. The genes in cluster C2 (*clsA*, *clsC* and *pgsA*) were enriched in the phospholipid biosynthesis pathway (KW-0594) and responsible for synthesis of cardiolipin and phosphatidylglycerol synthesis, whose loss could be compensated for by other anionic phospholipids (53). The genes in cluster C3 were deemed essential for MDR phenotype, and the associations among PhoPQ, CpxAR as well as global regulators were found in the C3 extended network, which indicated a coupling of signaling of CpxAR or PhoPQ in modulating the global regulators and relevant genes of the efflux pump and OM porin. Unfortunately, no interaction links were found between *glnGL* and ARGs. Previous studies reported that glutamine was responsible for synthesis of a poly-a-l-glutamine layer associated with the cell wall, and inhibition of GS activity enhanced the susceptibility to penicillin in Streptococcus pneumoniae and methicillin in Staphylococcus aureus (54, 55). In addition, the E. coli TCS GlnGL could govern transcription of *relA*/*spoT* genes and thereby led to elevated level of guanosine tetra- and pentaphosphate (ppGpp). In Vibrio cholerae, accumulation of ppGpp led to a suppressed central metabolism and iron transport system, resulting in reduced oxidative stress and increased antibiotic resistance (tetracycline, erythromycin, and chloramphenicol) (56, 57). Altogether, we speculated that *S*. Enteritidis GlnGL could activate expression of GS and ppGpp, which were involved in production of cell envelope constituents and regulation of intracellular redox balance, therefore conferring antibiotic resistance.

The regulatory effects of CpxAR and PhoPQ on multiple ARGs were demonstrated in this study. Bacteria could survive antibiotic exposure by expelling antibiotics through efflux pumps and blocking OM porin-mediated antibiotic influx (58). The global regulators RamA, SoxS, and MarA could modulate expression of efflux pumps and OM porins in Salmonella (59, 60). In this work, the absence of *cpxR*/*phoP* caused an increase in the expression level of OM porins and in membrane permeability as well as a decrease in expression level of specific efflux pumps and their regulatory factors, resulting in influx of a quantity of antibiotic molecules. Previous studies showed that the expression of OM porins STM1530 and OmpD was regulated by CpxAR to promote ceftriaxone resistance of *S.* Typhimurium (61). In E. coli, overproduction of CpxR was found to confer resistance to β-lactams in an *acrB*-free background (40). P. aeruginosa CpxAR was found to enhance quinolone resistance by activating expression of the *mexAB-oprM* efflux pump, while the absence of CpxR led to a Klebsiella pneumoniae phenotype susceptible to β-lactams and chloramphenicol (24, 62). Therefore, the regulatory function of CpxAR on efflux pumps and OM porins was universal across different species. On the other hand, it was proposed that PhoPQ could activate the putative ABC transporters in P. aeruginosa to extrude intracellular tetracycline (63), and the MdtK pump could expel ciprofloxacin into the periplasmic space in E. coli (64). To our knowledge, there were no direct clues to the resistance regulatory function of PhoPQ in *S*. Enteritidis. This study clarified the correlation between PhoPQ and efflux pumps by demonstrating the upregulatory effect of PhoPQ on the transcription level of the ABC-type family and MATE family pump genes (*macB* and *mdtK*). In addition, PhoP was reported as a transcriptional activator of *crp* in Yersinia pestis (65) and the loss of *crp* in *S.* Typhimurium could enhance resistance to fluoroquinolones by reducing permeability and elevating efflux of fluoroquinolones (33). Given the increased level of expression of *crp* in Δ*phoP* cells, we proposed that the absence of PhoP could derepress expression of *crp* and affect the function of multiple ARGs. In any case, CpxAR and PhoPQ in *S*. Enteritidis could modulate expression of the ARGs that encoded OM porins, efflux pumps, and global regulators, conferring resistance to quinolones and cephalosporins.

Previous studies proposed that there was a feedback mechanism employed to regulate expression of AcrAB-TolC and other functional efflux pumps by RamA and MarA (30, 60). Accordingly, a variety of compensatory pathways that improve fitness under the subinhibitory antibiotic pressure were presented in this study. Activation of EmrA pump in the absence of *phoP* or *cpxR* cells could be attributed to this feedback mechanism, which compensated for the loss of other pumps’ efflux function. In addition, it has been demonstrated that CpxR might regulate the colistin susceptibility of *S.* Typhimurium through PmrAB and PhoPQ (66). In this work, CpxR and PhoP showed cross-interaction in gene interaction networks, and qRT-PCR analysis further confirmed a compensatory expression of these two regulator genes, indicating a partial functional redundancy between PhoPQ and CpxAR. Nevertheless, these fitness mechanisms were not sufficient to completely make up for the sensitive phenotype caused by *phoP*/*cpxR* deletion, despite the adaptation of the Δ*phoP* and Δ*cpxR* mutants to antibiotics.

In conclusion, three TCSs, CpxAR, PhoPQ, and GlnGL, were identified as important regulators for MDR formation in *S*. Enteritidis. Deficiency in each of these TCSs results in a significantly reduced resistance to at least three classes of antibiotics, and this change was universal in *S*. Enteritidis. CpxAR and PhoPQ conferred resistance to quinolones and cephalosporins by altering expression of the genes responsible for drug efflux and drug entry, as well as those that caused significant changes in the global regulators. These TCSs were therefore considered potential targets in synergistic antibacterial therapy of MDR *S*. Enteritidis. In-depth characterization of crucial ones (e.g., GlnGL) that operate through gene regulation to uncover multiple cellular pathways for MDR is ongoing in our laboratory.

## MATERIALS AND METHODS

### Bacterial strains.

*S*. Enteritidis isolates (*n* = 7) were recovered from stool samples of patients with diarrhea during 2007 to 2017 in Shanghai and Henan, China, that were provided by the Shanghai Municipal Center for Disease Control and Prevention and reported in our recent papers (28, 67). These isolates belonged to ST11 (the predominant ST in *S*. Enteritidis) and exhibited the frequent antimicrobial resistance profile of AMP-SXT-NAL-CAZ-CRO-CTX-FEP (see Table S1 in the supplemental material), among which *S*. Enteritidis SJTUF12367 had the widest drug resistance profile (ACSSuT-NAL-KAN-CAZ-CRO-CTX-ceftiofur [TIO]-FEP-fosfomycin [FOS]). These strains were cultivated in lysogeny broth (LB) or on LB agar supplemented with or without appropriate antibiotics.

### Construction of gene deletion and complementation strains.

The TCS elements of the SJTUF12367 genome were identified via HMMER software and the Pfam database (Table S3 and Table S4). TCS gene deletion and complementation were performed as described previously (68). Briefly, TCS genes were first replaced by an FRT site-flanked *hph* cassette, which was a hygromycin resistance gene that was used as the resistance selection marker to construct homologous recombinant fragments and conferred resistance to hygromycin of TCS deletion strains. The cassette was then eliminated via Flp-FRT recombination to obtain markerless in-frame indel mutants. For the construction of complementary strains, the full-length regulator protein gene and its ribosome binding site were cloned into the pBAD33 vector with an arabinose-inducible promoter and then transformed into the corresponding mutants. The resulting strains were confirmed by PCR and DNA sequencing. The plasmids and primers used are shown in Table S1 and Table S5, respectively.

10.1128/msphere.00383-22.7 TABLE S3 Putative 33 HKs and 35 RRs encoded in *S*. Enteritidis SJTUF12367. blastp was performed on the whole-genome sequence of SJTUF12367 and the Pfam database (http://pfam.xfam.org) using the HMMER software suite. Proteins equipped with HisKA (PF00512), HisKA-2 (PF07568), HisKA-3 (PF07730), His-kinase (PF06580), HWE-HK (PF07536), H-Kinase-dim (PF02895), HisK-N (PF09385) or Hpt (PF01627), and HATPase-c (PF02518) were assumed as hypothetical histidine kinase (HK) elements, while proteins containing the functional domain of Response-Reg (PF00072) were assumed as hypothetical response regulator (RR) elements. The threshold values of HisKA and Hpt domains were set as an E value of <1, while the threshold values of HATPase-c and Response-reg domains were adjusted to an E value of <1e−5. Download Table S3, DOCX file, 0.03 MB. Copyright © 2022 Hu et al. 2022 Hu et al. https://creativecommons.org/licenses/by/4.0/ This content is distributed under the terms of the Creative Commons Attribution 4.0 International license.

10.1128/msphere.00383-22.8 TABLE S4 Homologs and putative functions of TCSs. The SMART tool (http://smart.embl-heidelberg.de/) was used to investigate the modular architecture of TCS elements. TM, transmembrane domain. Download Table S4, DOCX file, 0.02 MB. Copyright © 2022 Hu et al. 2022 Hu et al. https://creativecommons.org/licenses/by/4.0/ This content is distributed under the terms of the Creative Commons Attribution 4.0 International license.

### Antimicrobial susceptibility tests.

Antimicrobial susceptibility testing was conducted on *S*. Enteritidis isolates using the standard agar dilution method recommended by the CLSI (69). The antibiotics used included third- and fourth-generation (broad-spectrum) cephalosporins (ceftazidime, ceftriaxone, cefotaxime, ceftiofur and cefepime) as well as nalidixic acid, ofloxacin, ciprofloxacin, trimethoprim-sulfamethoxazole, ampicillin, gentamicin, streptomycin, kanamycin, azithromycin, tetracycline, and chloramphenicol. All antibiotics were purchased from Sigma, Inc. E. coli ATCC 25922 was used as a quality control for the MIC value determination.

### Construction of the gene interaction network.

ARGs of the SJTUF12367 genome were collected from three databases, including the Pathway-systems Resource Investigation Center (PATRIC; https://www.patricbrc.org/), Comprehensive Antibiotic Resistance Database (CARD; https://card.mcmaster.ca/), and National Database of Antibiotic Resistance Organisms (NDARO; https://www.ncbi.nlm.nih.gov/pathogens/antimicrobial-resistance/) (70). The interacting partner data were curated from the STRING database with the medium confidence scores (more than 0.4). The gene interaction network was visualized by Cytoscape. The Cytoscape tools MCODE and NetworkAnalyzer were used for identification of the highly interacting nodes (clusters) and computing the topological parameters for networks, respectively.

### Membrane perturbation assays.

The 1-*N*-phenylnaphthylamine (NPN) uptake assay was performed to determine the outer membrane permeability (36). NPN was added to the culture containing 2 × 10^6^ CFU/mL S. Enteritidis wild-type and Δ*cpxR*/Δ*phoP* cells (final NPN concentration of 12.5 μM) and incubated for 15 min with various concentrations of antibiotics. The fluorescence emission intensity was recorded (λ_exc_ = 340 nm; λ_em_ = 420 nm; gap width, 1 mm) using an Infinite 200 PRO microplate reader (Tecan, Switzerland).

### RNA isolation and qRT-PCR assays.

The cultures of exponentially growing cells were equalized to an optical density at 600 nm (OD_600_) value of 0.2. Volumes of 160-μL bacterial suspensions were then added to 3.84 mL LB, to which had been preadded subinhibitory concentrations of antibiotics to reach final concentrations of 32 mg/L (1/8 MIC) nalidixic acid (NAL) and 2 mg/L (1/16 MIC) ceftazidime (CAZ) (Fig. S4) (33). Samples were taken after incubation at 2 h and 8/10 h. RNA was extracted using TRIzol reagent (Invitrogen) as described by Huang et al. (48). Removal of residual genomic DNA and cDNA synthesis was conducted using the PrimeScript RT reagent kit with gDNA Eraser (TaKaRa). RNA integrity was confirmed by electrophoresis. The primers used are shown in Table S5. qRT-PCR was run in triplicate and amplified using the TB green Premix *Ex Taq* II reagent (TaKaRa). The relative gene transcriptional level was determined using the threshold cycle (2^−ΔΔ^*^CT^*) method (71) and converted into the log_2_ value. The expression of 16S rRNA was used as an internal reference. qRT-PCR data analysis was undertaken by a two-way analysis of variance (ANOVA) and Holm-Sidak’s multiple-comparison test via the Prism8.0.1 program, and all points were normalized relative to the untreated WT at 2 h.

### Data availability.

The complete genome sequence of *S*. Enteritidis SJTUF12367 containing a chromosome (GenBank accession no. CP003200) and two plasmids (GenBank accession no. CP041177 and CP041178) with abundant ARGs was deposited in NCBI (GenBank assembly accession no. GCA_004323915.1).
