# Supplementary figures and images for: Characterization of the Role of Two-Component Systems in Antibiotic Resistance Formation in Salmonella enterica Serovar Enteritidis
Source: mSphere. 2022 Oct 26;7(6):e00383-22. doi: 10.1128/msphere.00383-22 (PMC9769886; doi:10.1128/msphere.00383-22)

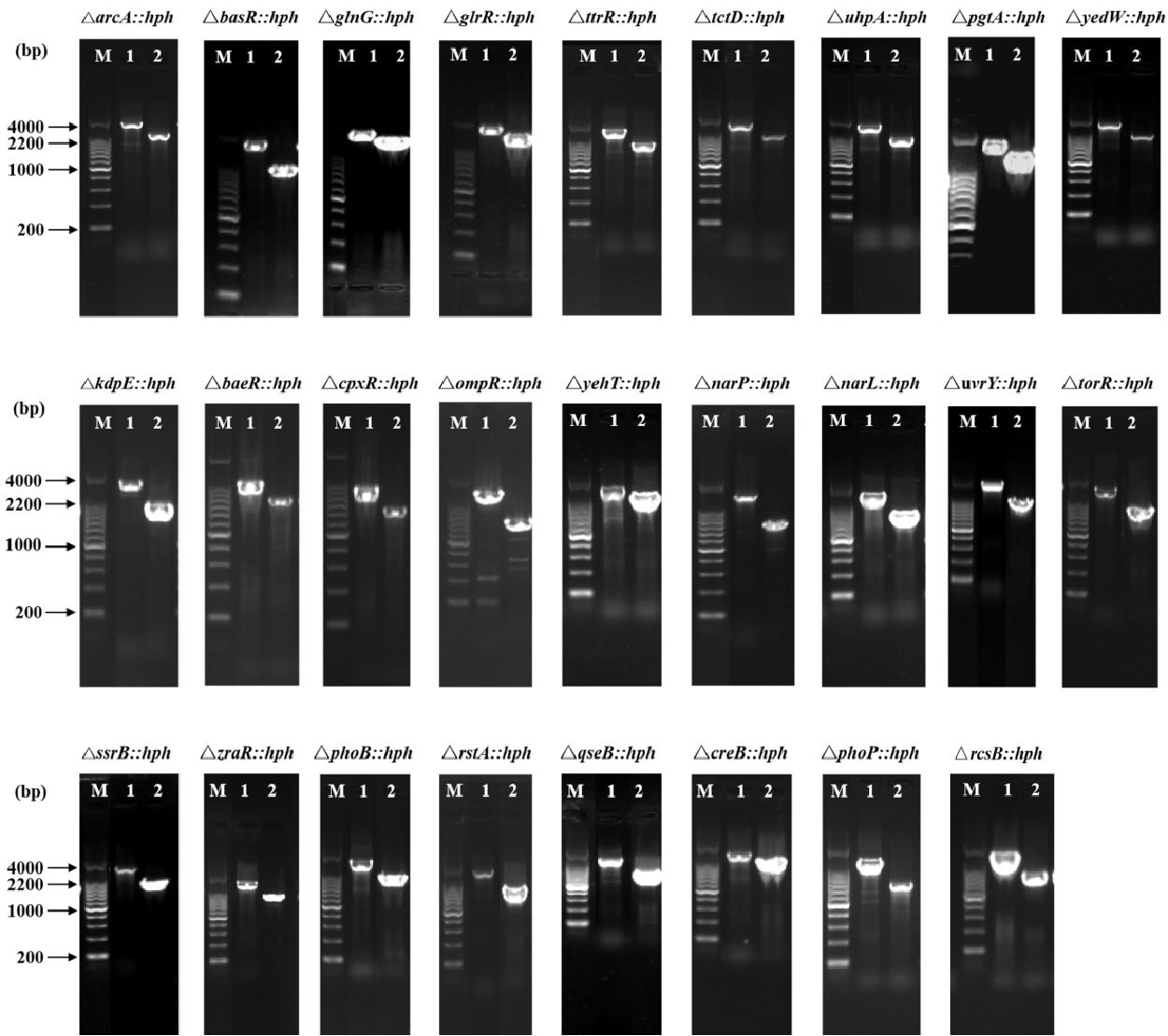

Supplement: FIG S1 [file msphere.00383-22-s0001.tif]

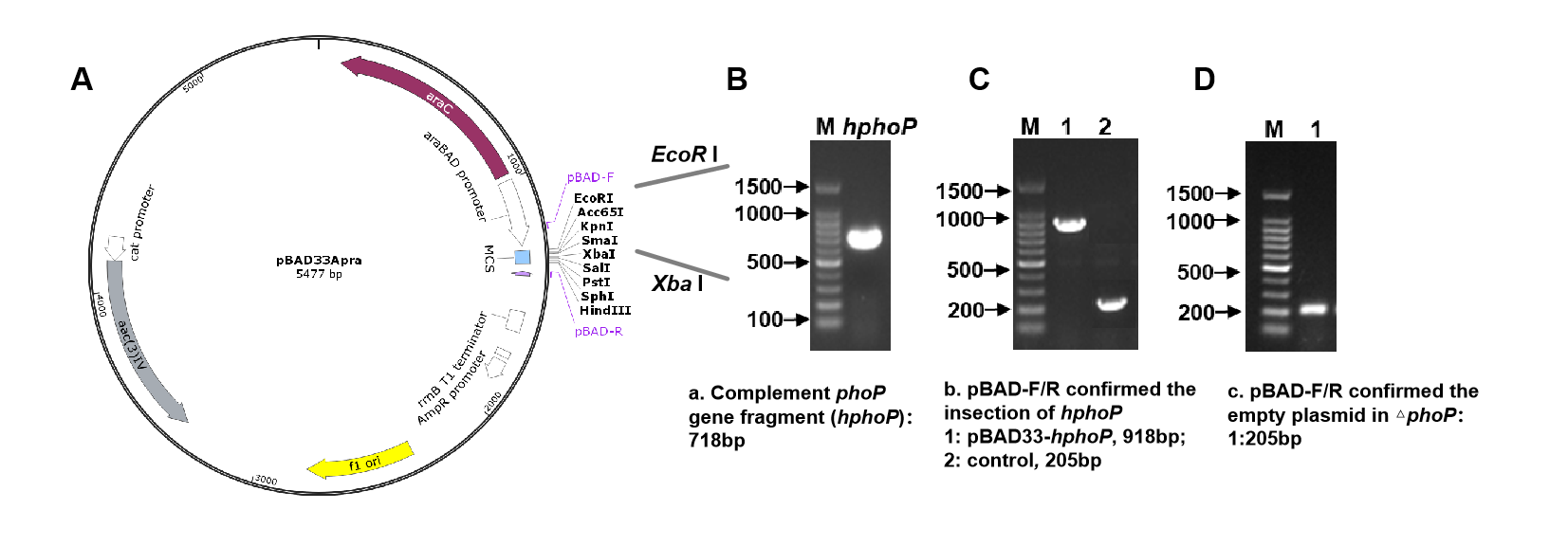

Supplement: FIG S2 [file msphere.00383-22-s0002.tif]

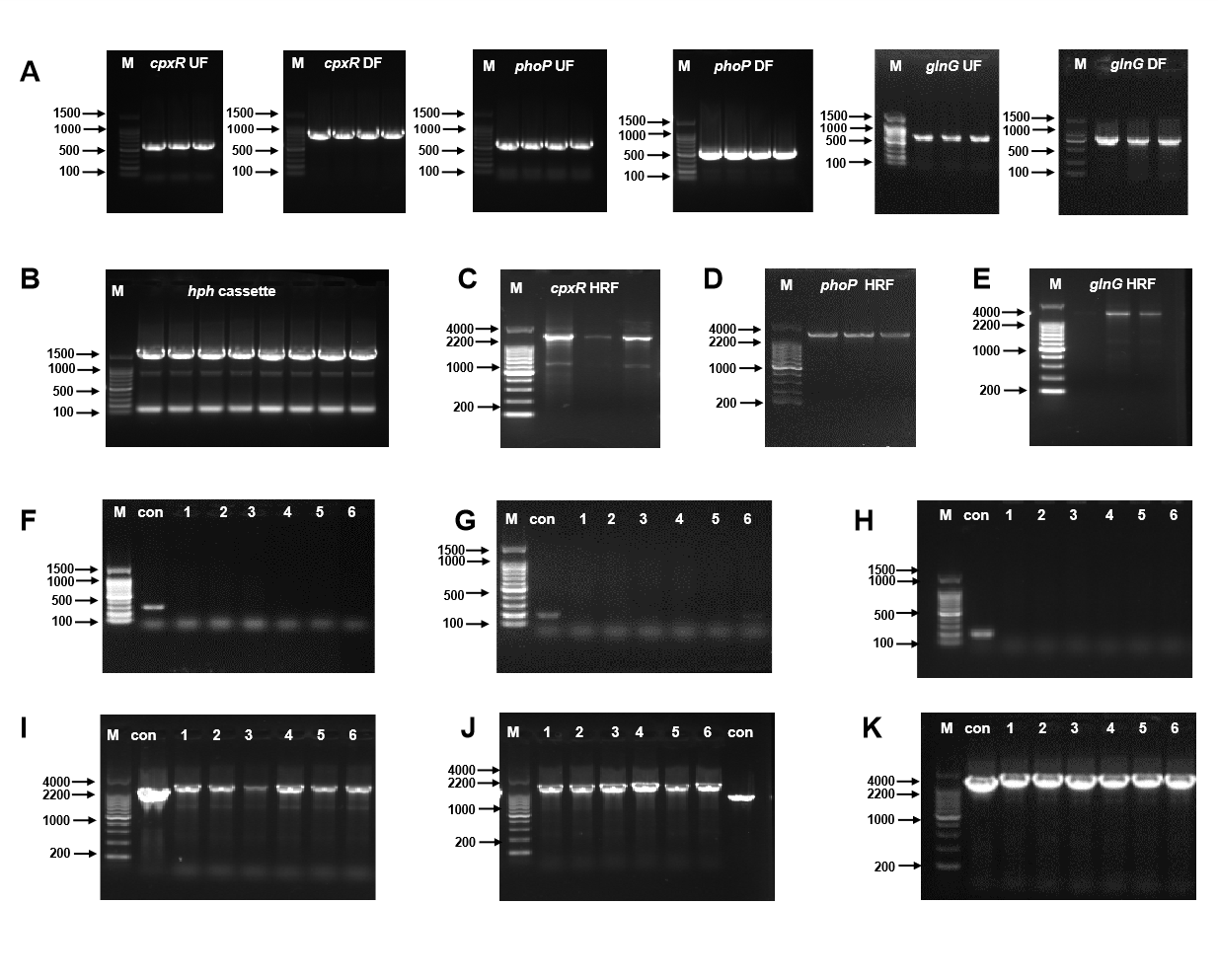

Supplement: FIG S3 [file msphere.00383-22-s0003.tif]

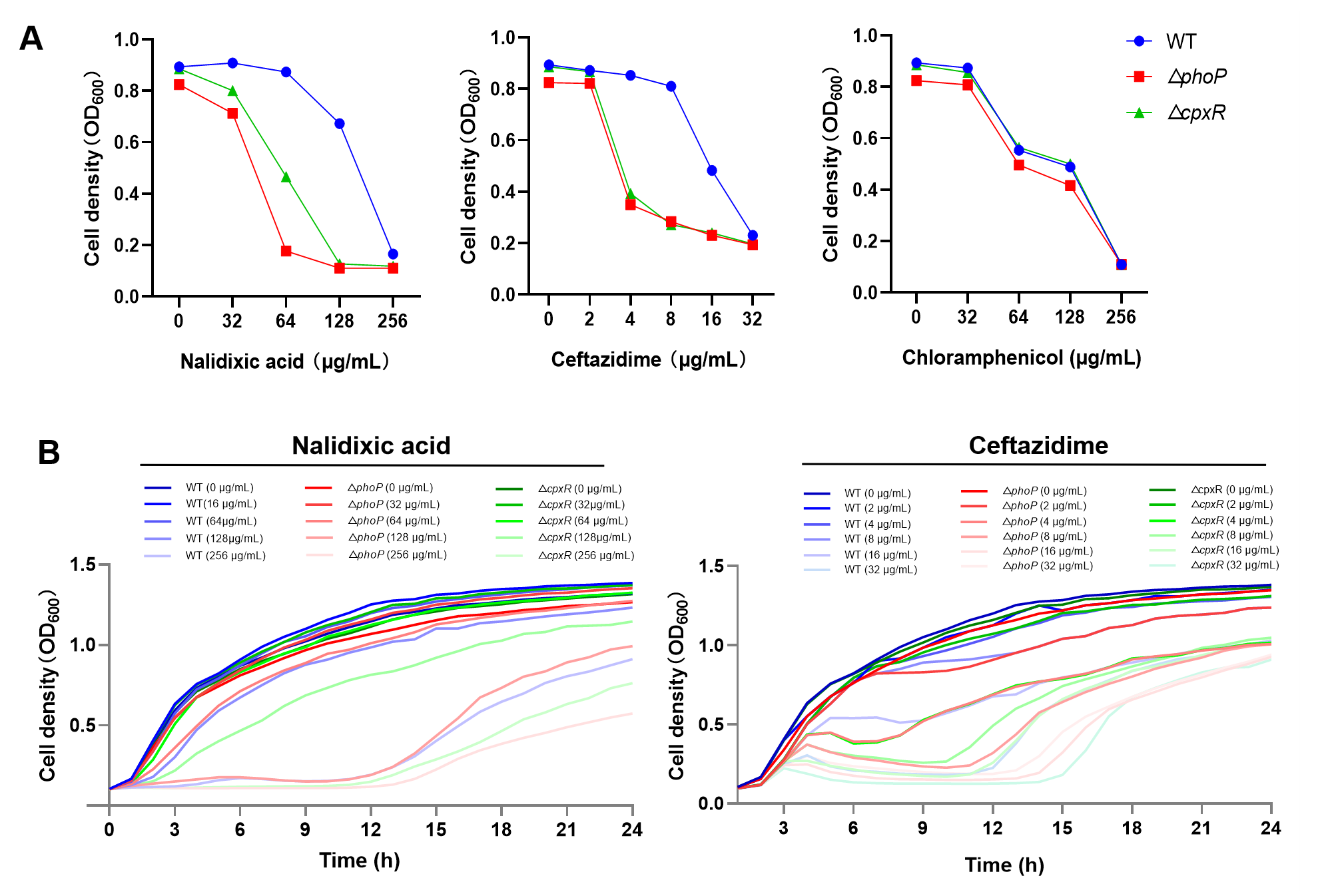

Supplement: FIG S4 [file msphere.00383-22-s0004.tif]
