# Supplementary material for: Characterization of the Role of Two-Component Systems in Antibiotic Resistance Formation in Salmonella enterica Serovar Enteritidis
Source: mSphere. 2022 Oct 26;7(6):e00383-22. doi: 10.1128/msphere.00383-22 (PMC9769886; doi:10.1128/msphere.00383-22)
Supplement: TABLE S5 [file msphere.00383-22-s0009.docx]

**Table S5. Primary primers used for mutagenesis and qRT-PCR.**

| **Primer** | **Sequence (5′ to 3′)** | **Target** |
| --- | --- | --- |
| HphF560M | agctcggtaccaattgggga | pUC19-Hyg: *hph* cassette:  1686 bp |
| HphR1180 | cctgcaggtcgactctagaggat |  |
| glnG_UF-F | tgcccgacccggcgctaa | Upstream flank of *glnG* UF:  588 bp |
| glnG_UF-R | tacttcaagatccccaattggtaccgagcttgcataaacacctctatttccga |  |
| glnG_DF-F | attcgggatcctctagagtcgacctgcagggttgaaagagctgggaatggag | Downstream flank of *glnG* DF:  505bp |
| glnG_DF-R | gatttgcagttactgtcgccgc |  |
| glnG_UJ-F | tgatgcgcgaaagtttagcgg | Amplification and verification of UF*+glnG+*DF gene fragment: 2934 bp |
| glnG_DJ-R | tgaagcgttattaccagcaagtg |  |
| glnG_In-F | cattattggtcggctgtcgc | The fragment of *glnG*:  168 bp |
| glnG_In-R | tcggattcaatcaaatctttcg |  |
| phoP_UF-F | tctaacgcagtgttgcaccatct | Upstream flank of *phoP* UF:  594 bp |
| phoP_UF-R | tacttcaagatccccaattggtaccgagctaaccagtacgcgcatcatctctt |  |
| phoP_DF-F | attcgggatcctctagagtcgacctgcaggccgcacgatgtcattaccaccg | Downstream flank of *phoP* DF:  528 bp |
| phoP_DF-R | cacgtacttctttgagtttttcctg |  |
| phoP_UJ-F | tcaaccgtcctgcgtaacct | Amplification and verification of UF*+phoP+*DF gene fragment: 1522 bp |
| phoP_DJ-R | gtttcaatttcatggaagccgt |  |
| phoP_In-F | aaggctggcaggataaagtcg | The fragment of *phoP*:  153 bp |
| phoP_In-R | ggaacggcgggatgttga |  |
| cpxR_UF-F | gcgcagttgctccattcgtt | Upstream flank of *cpxR* UF:  555 bp |
| cpxR_UF-R | tacttcaagatccccaattggtaccgagctacgtcatcaggcgtcgctaatc |  |
| cpxR_DF-F | attcgggatcctctagagtcgacctgcaggcgtggtcgcggctatctga | Downstream flank of *cpxR* DF:  565 bp |
| cpxR_DF-R | agcggcgaactgaccagca |  |
| cpxR_UJ-F | ccggctaaccagccgtccatagg | Amplification and verification of UF*+cpxR+*DF gene fragment: 2377 bp |
| cpxR_DJ-R | agacgctgcgcttcggtttcaat |  |
| cpxR_In-F | catcgatttacttttgcttgacg | The fragment of *cpxR*:  289 bp |
| cpxR_In-R | gcttagcgcatcgacttccag |  |
| EBGNHe-5 | cccgctagcgaaaagatgtttcgtgaagc | pKOBEG-Apra  ~1900 bp |
| EBGh3-3 | gggaagcttattatcgtgaggatgcgtca |  |
| PR1655 | tgctctagagcacggcattttcttttgcgttt | pFLP2-Apra  ~1600 bp |
| PR1656 | cgcggatccgcgtctttaggcccgtagtctgc |  |
| hglnG-F | ccggaattccggatccgggagagtacaagtatgcaacgaggaatagtctgggt | The *glnG* complement fragment：  1471bp |
| hglnG-R | gctctagagcctttacactcgcgggtaatgttt |  |
| hphoP-F | ccggaattccggtaacacaagggagaagagatgatgcgcgtactggttgtag | The *phoP* complement fragment：  718 bp |
| hphoP-R | gctctagagctcattagcgcaattcaaaaaga |  |
| hcpxR-F | ccggaattccggctcggaggtacgtaaacaatgaataaaatcctgttagttga | The *cpxR* complement fragment：  809 bp |
| hcpxR-R | gctctagagcagtaccagcattagcacca |  |
| pBAD-F | atgccatagcatttttatcc | pBAD33-Apra:  205 bp |
| pBAD-R | gatttaatctgtatcagg |  |
| 16SrDNA-F | cgaattaaaccacatgctccac | internal reference |
| 16SrDNA-R | ctggtagtccacgccgtaaac |  |
| acrA-F | gaaaacggcaaagcgaagg | RND efflux pump |
| acrA-R | ccgggttagggaagatggc |  |
| acrD-F | tgtttgttcgcttgaaagactgg |  |
| acrD-R | acgcgagcttctttaatctggt |  |
| mdsA-F | accccgacggaatgctt |  |
| mdsA-R | cggacgatactccacctga |  |
| emrA-F | tccaaaccccgcagcaac | major facilitator superfamily pump |
| emrA-R | gccacgtaagcatcatctgtct |  |
| mdtK-F | cgcgtcagttattagcgttagc | multidrug and toxic compound extrusion family pump |
| mdtK-R | ggcgggaagccagatagagg |  |
| macB-F | gcttacccgcctgctcacc | ATP binding cassette family pump |
| macB-R | gccgacaaccagcgaaatg |  |
| Crp-F | tcttgtctcattgccacattcata | global regulators |
| Crp-R | gcactgccacggagccttt |  |
| marA-F | gtccagacgcaacactgacgct |  |
| marA-R | catttggaatatcctgaacgctc |  |
| soxS-F | gctaaacattgatgtggtggca |  |
| soxS-R | ggagacggcgctggcgaa |  |
| ramA-F | gaccatttccgctcaggttatc |  |
| ramA-R | ccgccagttttagcttccgt |  |
| ompA-F | tcacccctgaaatcgcaac | outer membrane porins |
| ompA-R | gccgaaacggtaggaaaca |  |
| ompF-F | tctgacttctttggtctggtgg |  |
| ompF-R | gcttgctgttgctgtacgctg |  |
| OmpD-F | ccatcgcttacctgaaatccaa |  |
| OmpD-R | ggtgaagtcgctgtcgtcc |  |
| OmpC-F | cttcggcctggtggatgg |  |
| OmpC-R | gcgaccgttggtgttttca |  |
