# Supplementary material for: Characterization of the Role of Two-Component Systems in Antibiotic Resistance Formation in Salmonella enterica Serovar Enteritidis
Source: mSphere. 2022 Oct 26;7(6):e00383-22. doi: 10.1128/msphere.00383-22 (PMC9769886; doi:10.1128/msphere.00383-22)
Supplement: TABLE S1 [file msphere.00383-22-s0005.docx]

**Table S1. [Primary](D:/Program%20Files%20(x86)/Youdao/Dict/8.9.9.0/resultui/html/index.html#/javascript:;) strains and plasmids used in this study.**

| **Strains (*S.* Enteritidis)** | **Descriptions** |
| --- | --- |
| SJTUF 12367 | ACSSuT-FOS-KNA-CAZ-CRO-CTX-TIO-FEP-CEF-NAL |
| Δ*arcA*::*hph*, Δ*basR*::*hph*, Δ*glrR*::*hph*, Δ*ttrR*::*hph*, Δ*tctD*::*hph*, Δ*uhpA*::*hph*, Δ*yedW*::*hph*, Δ*pgtA*::*hph*, Δ*kdpE*::*hph*, Δ*baeR*::*hph*, Δ*ompR*::*hph*, Δ*yehT*::*hph*, Δ*narP*::*hph*, Δ*uvrY*::*hph*, Δ*torR*::*hph*, Δ*narL*::*hph*, Δ*ssrB*::*hph*, Δ*zraR*::*hph*, Δ*phoB*::*hph*, Δ*rstA*::*hph*, Δ*qseB*::*hph*, Δ*creB*::*hph*, Δ*rcsD*::*hph*, Δ*glnG*::*hph*, Δ*cpxR*::*hph*, Δ*phoP*::*hph* | Twenty-six TCS mutants with *hph* cassette marker |
| Δ*cpxR*,Δ*glnG*,Δ*phoP* | *hph* cassette-free mutant strains |
| Δ*cpxR*::*cpxR*,Δ*glnG*::*glnG*,Δ*phoP*::*phoP* | Complementary strains |
| SJTUF 14364 | ACSSuT-KNA-CAZ-CRO-CTX-TIO-FEP-NAL |
| Δ*cpxR*::*hph*,Δ*glnG*::*hph*,Δ*phoP*::*hph* | TCS mutants with *hph* marker |
| SJTUF14365 | ACSSuT-KNA-CAZ-CRO-CTX-TIO-FEP-NAL |
| Δ*cpxR*::*hph*,Δ*glnG*::*hph*,Δ*phoP*::*hph* | TCS mutants with *hph* marker |
| SJTUF 14409 | AMP-SXT-CAZ-KNA-CRO-CTX-TIO-FEP-NAL |
| Δ*cpxR*::*hph*,Δ*glnG*::*hph*,Δ*phoP*::*hph* | TCS mutants with *hph* marker |
| SJTUF 14745 | AMP-SXT-CAZ-CRO-CTX-TIO-FEP-NAL |
| Δ*cpxR*::*hph*,Δ*glnG*::*hph*,Δ*phoP*::*hph* | TCS mutants with *hph* marker |
| SJTUF 14749 | ACSSuT-KNA-CAZ-CRO-CTX-TIO-FEP-NAL |
| Δ*cpxR*::*hph*,Δ*glnG*::*hph*,Δ*phoP*::*hph* | TCS mutants with *hph* marker |
| SJTUF 14751 | ACSSuT-KNA-CAZ-CRO-CTX-TIO-FEP-NAL |
| Δ*cpxR*::*hph*,Δ*glnG*::*hph*,Δ*phoP*::*hph* | TCS mutants with *hph* marker |
| **Plasmid** | **Descriptions** |
| pKOBEG-Apra | The plasmid is thermo-sensitive and apramycin-resistant, and contains arabinose-inducible lambda red genes *gam*, *bet* and *exo*. This is a helper plasmid for mutagenesis. |
| pUC19-Hyg | The plasmid is derived from pUC19. It contains a flippase recognition target (FRT)-flanked *hph* cassette, which confers resistance to hygromycin. |
| pFLP2-Apra | The plasmid is derived from pFLP2 with the *bla* replaced by an apramycin-resistant gene, that is used to flip out the *hph* cassette. |
| pBAD33-Apra | The plasmid is derived from pBAD33 with the *cat* replaced by an apramycin-resistant gene. It contains an arabinose-inducible vector and is used for construction of compensatory strain. |
