# Supplementary material for: Characterization of the Role of Two-Component Systems in Antibiotic Resistance Formation in Salmonella enterica Serovar Enteritidis
Source: mSphere. 2022 Oct 26;7(6):e00383-22. doi: 10.1128/msphere.00383-22 (PMC9769886; doi:10.1128/msphere.00383-22)
Supplement: TABLE S2 [file msphere.00383-22-s0006.docx]

**Table S2. Functional enrichment of C3 extend network genes**

| **Category** | **term ID** | **Description** | **Matching proteins in C3 extend network** |
| --- | --- | --- | --- |
| GO Process | GO:0042255 | Ribosome assembly | *rplQ*,*rpsD*,*rpsK*,*rpsE*,*rplF*,*rpsH*,*rplE*,*rplX*,*rpsQ*,*rplP*,*rpsC*,*rplV* |
|  | GO:0046677 | Response to antibiotic | *acrA*,*macA*,*macB*,*marR*,*acrD*,*emrA*,*emrB*,*tolC*,*rpsD*,*rpsE*,*rplF*,*rpsQ*,*rplV* |
|  | GO:0010467 | Gene expression | *rpoD*,*rplQ*,*rpoA*,*rpsD*,*rpsK*,*rpsM*,*rplO*,*rpsE*,*rplR*,*rplF*,*rpsH*,*rplE*,*rplX*,*rplN*,*rpsQ*,*rplP*,*rpsC*,*rplV*,*crp* |
|  | GO:0042908 | Xenobiotic transport | *acrB*,*acrA*,*macA*,*macB*,*acrD*,*emrA*,*emrB*,*tolC* |
|  | GO:0042221 | Response to chemical | *acrA*,*macA*,*macB*,*phoQ*,*marR*,*mgrB*,*acrD*,*emrA*,*emrB*,*tolC*,*rpsD*,*rpsE*,*rplF*,*rpsQ*,*rplV*,*crp* |
|  | GO:0044260 | Cellular macromolecule metabolic process | ,*phoQ*,*rpoD*,,*rplQ*,*rpoA*,*rpsD*,*rpsK*,*rpsM*,*rplO*,*rpsE*,*rplR*,*rplF*,*rpsH*,*rplE*,*rplX*,*rplN*,*rpsQ*,*rplP*,*rpsC*,*rplV*,*crp*,*cpxA* |
|  | GO:1901566 | Organonitrogen compound biosynthetic process | *rplQ*,*rpsD*,*rpsK*,*rpsM*,*rplO*,*rpsE*,*rplR*,*rplF*,*rpsH*,*rplE*,*rplX*,*rplN*,*rpsQ*,*rplP*,*rpsC*,*rplV*,*cyaA* |
|  | GO:0050896 | Response to stimulus | *acrA*,*macA*,*macB*,*phoQ*,*phoP*,*marR*,*mgrB*,*acrD*,*emrA*,*emrB*,*tolC*,*rpoD*,*rpsD*,*rpsE*,*rplF*,*rpsQ*,*rplV*,*crp*,*cpxA*,*cpxR* |
|  | GO:0050794 | Regulation of cellular process | *phoQ*,*phoP*,*marR*,*mgrB*,*rpoD*,,*rpsD*,*rpsH*,*crp*,*cpxA*,*cpxR*,*cytR* |
|  | GO:0051252 | Regulation of rna metabolic process | *phoP*,*marR*,*rpoD*,,*rpsD*,*rpsH*,*crp*,*cpxR*,*cytR* |
|  | GO:0006807 | Nitrogen compound metabolic process | *phoQ*,*rpoD*,*rplQ*,*rpoA*,*rpsD*,*rpsK*,*rpsM*,*rplO*,*rpsE*,*rplR*,*rplF*,*rpsH*,*rplE*,*rplX*,*rplN*,*rpsQ*,*rplP*,*rpsC*,*rplV*,*crp*,*cyaA*,*cpxA* |
|  | GO:0007154 | Cell communication | *phoQ*,*phoP*,*crp*,*cpxA*,*cpxR* |
|  | GO:0010468 | Regulation of gene expression | *phoP*,*marR*,*rpoD*,,*rpsD*,*rpsH*,*crp*,*cpxR*,*cytR* |
| GO Function | GO:0003723 | RNA binding | *rpsD*,*rpsK*,*rpsM*,*rplO*,*rpsE*,*rplR*,*rplF*,*rpsH*,*rplE*,*rplX*,*rplN*,*rpsQ*,*rplP*,*rpsC*,*rplV* |
|  | GO:0003676 | Nucleic acid binding | *phoP*,*marR*,*rpoD*,,*rpoA*,*rpsD*,*rpsK*,*rpsM*,*rplO*,*rpsE*,*rplR*,*rplF*,*rpsH*,*rplE*,*rplX*,*rplN*,*rpsQ*,*rplP*,*rpsC*,*rplV*,*crp*,*cpxR*,*cytR* |
|  | GO:0015562 | Efflux transmembrane transporter activity | *acrB*,*macA*,*macB*,*acrD*,*tolC* |
| GO Component | GO:0032991 | Protein-containing complex | *acrB*,*acrA*,*macA*,*macB*,*phoP*,*tolC*,*rplQ*,*rpsD*,*rpsK*,*rpsM*,*rplO*,*rpsE*,*rplR*,*rplF*,*rpsH*,*rplE*,*rplX*,*rplN*,*rpsQ*,*rplP*,*rpsC*,*rplV*,*crp*,*cpxR* |
|  | GO:1990281 | Efflux pump complex | *acrB*,*acrA*,*macA*,*tolC* |
|  | GO:1902495 | Transmembrane transporter complex | *acrB*,*acrA*,*macA*,*macB*,*tolC* |
| STRING clusters | CL:46 | Ribosome | *rplQ*,*rpoA*,*rpsD*,*rpsK*,*rpsM*,*rplO*,*rpsE*,*rplR*,*rplF*,*rpsH*,*rplE*,*rplX*,*rplN*,*rpsQ*,*rplP*,*rpsC*,*rplV* |
|  | CL:3574 | Efflux pump complex, and multidrug efflux pump-associated protein acrz | *acrB*,*acrA*,*macA*,*macB*,*marR*,*acrD*,*emrA*,*emrB*,*tolC* |
| KEGG | eco01503 | Cationic antimicrobial peptide (CAMP) resistance | *acrB*,*acrA*,*phoQ*,*phoP*,*tolC*,*cpxA*,*cpxR* |
|  | eco02020 | Two-component system | *phoQ*,*phoP*,*acrD*,*tolC*,*crp*,*cpxA*,*cpxR* |
|  | eco01501 | beta-Lactam resistance | *acrB*,*acrA*,*tolC* |

False discovery rate <0.05.
