# Supplementary material for: Characterization of the Role of Two-Component Systems in Antibiotic Resistance Formation in Salmonella enterica Serovar Enteritidis
Source: mSphere. 2022 Oct 26;7(6):e00383-22. doi: 10.1128/msphere.00383-22 (PMC9769886; doi:10.1128/msphere.00383-22)
Supplement: TABLE S3 [file msphere.00383-22-s0007.docx]

**Table S3. Putative 33 HKs and 35 RRs encoded in *S.* Enteritidis SJTUF12367**

| **ORF(Sequence)** | **Class** | **Trans-**  **membrane** | **Homologs** | **Length(aa)** | **Conserved domain** | | | | | | |
| --- | --- | --- | --- | --- | --- | --- | --- | --- | --- | --- | --- |
|  |  |  |  |  | **H-kinase_dim** | **Hpt** | **HisKA** | **Response_reg** | **HATPase_c** | **HisKA_3** | **His_kinase** |
| WP_000216812.1 | HK | 2 | NarQ | 566 |  |  |  |  | y | y |  |
| WP_000476244.1 | HK | 2 | NarX | 598 |  |  |  |  | y | y |  |
| WP_001091262.1 | HK | 7 | UhpB | 500 |  |  |  |  | y | y |  |
| WP_000061323.1 | HK | 0 | CheA | 671 | y | y |  |  | y |  |  |
| WP_000186390.1 | HK | 2 | BarA | 918 |  | y | y | y | y |  |  |
| WP_000083201.1 | HK | 2 | RcsD | 889 |  | y |  |  | y |  |  |
| WP_000809819.1 | HK | 2 | ArcB | 778 |  | y | y | y | y |  |  |
| WP_000106946.1 | HK | 1 | TorS | 911 |  | y | y | y | y |  |  |
| WP_001050774.1 | RR | 1 | SsrA | 920 |  | y | y | y | y |  |  |
| WP_000876084.1 | HK | 2 | RcsC | 948 |  |  | y | y | y |  |  |
| WP_001680270.1 | HK | 2 | ZraS | 465 |  |  | y |  | y |  |  |
| WP_000893646.1 | HK | 1 | PhoR | 431 |  |  | y |  | y |  |  |
| WP_000997469.1 | HK | 3 | KdpD | 894 |  |  | y |  | y |  |  |
| WP_000580402.1 | HK | 2 | CpxA | 457 |  |  | y |  | y |  |  |
| WP_000946040.1 | HK | 0 | CusS | 109 |  |  |  |  | y |  |  |
| WP_001253818.1 | HK | 2 | EnvZ | 450 |  |  | y |  | y |  |  |
| WP_001212189.1 | HK | 2 | BasS | 356 |  |  | y |  | y |  |  |
| WP_000870073.1 | HK | 2 | BaeS | 467 |  |  | y |  | y |  |  |
| WP_000732946.1 | HK | 2 | RstB | 433 |  |  | y |  | y |  |  |
| WP_000682894.1 | HK | 2 | DcuS | 543 |  |  |  |  | y |  |  |
| WP_000146192.1 | HK | 0 | GlnL | 349 |  |  | y |  | y |  |  |
| WP_000779338.1 | HK | 2 | QseC | 449 |  |  | y |  | y |  |  |
| WP_001676035.1 | HK | 2 | QseE | 476 |  |  | y |  | y |  |  |
| WP_001219533.1 | HK | 2 | CreC | 474 |  |  | y |  | y |  |  |
| WP_001214411.1 | HK | 1 | TtrS | 592 |  |  | y |  | y |  |  |
| WP_000121823.1 | HK | 2 | DpiB | 553 |  |  |  |  | y |  |  |
| WP_001677995.1 | HK | 12 | CitA | 539 |  |  |  |  | y |  |  |
| WP_000872348.1 | HK | 2 | TctE | 471 |  |  | y |  | y |  |  |
| WP_000678486.1 | HK | 2 | PgtB | 668 |  |  | y |  | y |  |  |
| WP_000240033.1 | HK | 2 | YedV | 454 |  |  | y |  | y |  |  |
| WP_001031687.1 | HK | 2 | PhoQ | 487 |  |  | y |  | y |  |  |
| WP_001122550.1 | HK | 0 | MutL | 618 |  |  |  |  | y |  |  |
| WP_000272845.1 | HK | 6 | YehU | 561 |  |  |  |  | y |  | y |
| WP_000763861.1 | RR | 0 | CheY | 129 |  |  |  | y |  |  |  |
| WP_000611323.1 | RR | 0 | UvrY | 218 |  |  |  | y |  |  |  |
| WP_000625591.1 | RR | 0 | GlrR | 445 |  |  |  | y |  |  |  |
| WP_001188783.1 | RR | 0 | glnG | 469 |  |  |  | y |  |  |  |
| WP_000617928.1 | RR | 0 | ZraR | 441 |  |  |  | y |  |  |  |
| WP_001064598.1 | RR | 0 | NarL | 216 |  |  |  | y |  |  |  |
| WP_001157751.1 | RR | 0 | OmpR | 239 |  |  |  | y |  |  |  |
| WP_001033731.1 | RR | 0 | CpxR | 232 |  |  |  | y |  |  |  |
| WP_001115497.1 | RR | 0 | NarP | 215 |  |  |  | y |  |  |  |
| WP_000113921.1 | RR | 0 | PhoB | 229 |  |  |  | y |  |  |  |
| WP_000193432.1 | RR | 0 | RssB | 337 |  |  |  | y |  |  |  |
| WP_000186059.1 | RR | 0 | KdpE | 225 |  |  |  | y |  |  |  |
| WP_000801273.1 | RR | 0 | FimZ | 210 |  |  |  | y |  |  |  |
| WP_001194357.1 | RR | 0 | ArcA | 238 |  |  |  | y |  |  |  |
| WP_000698207.1 | RR | 0 | YedW | 226 |  |  |  | y |  |  |  |
| WP_000598637.1 | RR | 0 | YehT | 239 |  |  |  | y |  |  |  |
| WP_000697899.1 | RR | 0 | BasR | 222 |  |  |  | y |  |  |  |
| WP_001562512.1 | RR | 0 | TorR | 230 |  |  |  | y |  |  |  |
| WP_000930836.1 | RR | 0 | PgtA | 415 |  |  |  | y |  |  |  |
| WP_001187046.1 | RR | 0 | CreB | 229 |  |  |  | y |  |  |  |
| WP_001237934.1 | RR | 0 | TctD | 224 |  |  |  | y |  |  |  |
| WP_000036389.1 | RR | 0 | CheB | 349 |  |  |  | y |  |  |  |
| WP_000986522.1 | RR | 0 | PhoP | 224 |  |  |  | y |  |  |  |
| WP_001061919.1 | RR | 0 | RcsB | 216 |  |  |  | y |  |  |  |
| WP_000633672.1 | RR | 0 | UhpA | 196 |  |  |  | y |  |  |  |
| WP_000137854.1 | RR | 0 | BaeR | 240 |  |  |  | y |  |  |  |
| WP_000666335.1 | RR | 0 | SsrB | 212 |  |  |  | y |  |  |  |
| WP_001221574.1 | RR | 0 | QseB | 219 |  |  |  | y |  |  |  |
| WP_000190927.1 | RR | 0 | TtrR | 206 |  |  |  | y |  |  |  |
| WP_000138786.1 | RR | 0 | DpiA | 226 |  |  |  | y |  |  |  |
| WP_000377406.1 | RR | 0 | CitB | 228 |  |  |  | y |  |  |  |
| WP_000611307.1 | RR | 0 | DcuR | 239 |  |  |  | y |  |  |  |
| WP_001080048.1 | RR | 0 | RstA | 243 |  |  |  | y |  |  |  |
| WP_000368558.1 | RR | 0 | CheV | 333 |  |  |  | y |  |  |  |
| WP_001196089.1 | RR | 0 | YhjB | 200 |  |  |  | y |  |  |  |

HisKA, HK dimerization/phosphoaceptor domain; HATPase-c, HK-type ATPase catalytic domain; Hpt, histidine-containing phosphotransfer domain; H-Kinase-dim, HK homodimeric domain; HWE-HK, HWE histidine kinase.
