# Supplementary material for: Characterization of the Role of Two-Component Systems in Antibiotic Resistance Formation in Salmonella enterica Serovar Enteritidis
Source: mSphere. 2022 Oct 26;7(6):e00383-22. doi: 10.1128/msphere.00383-22 (PMC9769886; doi:10.1128/msphere.00383-22)
Supplement: TABLE S4 [file msphere.00383-22-s0008.docx]

**Table S4. Homologs and putative functions of TCSs**

| **Synonym** | **Homologs**  **(HK/RR)** | **Strand** | **HK domain arrangement^a^** | **RR domain arrangement** | **RR type** | **Putative function** |
| --- | --- | --- | --- | --- | --- | --- |
| WP_000216812.1/WP_001115497.1 | NarQ/NarP | _ | PilJ-HAMP-HisKA_3-HATPase_c | REC-HTH_LUXR | NarL | Nitrogen metabolism |
| WP_000476244.1/WP_001064598.1 | NarX/NarL | + | PilJ-TM-HAMP-HisKA_3-HATPase_c | REC-HTH_LUXR | NarL | Nitrate-induced regulation |
| WP_001091262.1/WP_000633672.1 | UhpB/UhpA | + | MASE1-HisKA_3-HATPase_c | REC-HTH_LUXR | NarL | Hexose phosphate transport |
| WP_000061323.1/WP_000763861.1 | CheA/CheY | _ | HPT-CheY_binding-H-kinase_dim  -HATPase_c-CheW | REC | CheB | Chemotaxis |
| /WP_000036389.1 | /CheB | _ |  | REC-CheB_methylest |  |  |
| /WP_001194357.1 | /CheV | _ |  | CheW-REC |  |  |
| WP_000186390.1/WP_000611323.1 | BarA/UvrY | _ | DUF2222-HAMP-HisKA-HATPase_c-REC-HPT | REC-HTH_LUXR | NarL | Regulate metabolism, motility, biofilm formation, stress resistance |
| WP_000809819.1/WP_001194357.1 | ArcB/ArcA | + | 2TM-PAS-HisKA-HATPase_c-REC-HPT | REC-Trans_reg_C | OmpR | Anaerobic respiration |
| WP_000106946.1/WP_001562512.1 | TorS/TorR | _ | TM-HAMP-HisKA-HATPase_c-REC-HPT | REC-Trans_reg_C | OmpR | Trimethylamine N-oxide respiration |
| WP_001050774.1/WP_000666335.1 | SsrA/SsrB | _ | TM-HAMP-HisKA-HATPase_c-REC | REC-HTH_LUXR | LuxR | Regulates SPI-2 |
| WP_000083201.1/WP_001061919.1 | RcsD/RcsB | _ | 2TM-HATPase_c-RcsD_ABL-HPT | REC-HTH_LUXR | NarL | Regulation of the synthesis of colonic acid capsule and motility |
| WP_000876084.1/ | RcsC/ | + | 2TM-PAS-HisKA-HATPase_c-RcsC-REC |  |  |  |
| WP_001680270.1/WP_000617928.1 | ZraS/ZraR | _ | 2TM-HisKA-HATPase_c | REC-AAA-HTH_8 | NtrC | Regulation of hydrogenase 3 formation |
| WP_000893646.1/WP_000113921.1 | PhoR/PhoB | _ | DUF3329-PAS-HisKA-HATPase_c | REC-Trans_reg_C | OmpR | Phosphate stress response |
| WP_000997469.1/WP_000186059.1 | KdpD/KdpE | + | KdpD-DUF4118-GAF_3-HisKA-HATPase_c | REC-Trans_reg_C | OmpR | Potassium transport |
| WP_000580402.1/WP_001033731.1 | CpxA/CpxR | + | TM-CpxA_peri-HAMP-HisKA-HATPase_c | REC-Trans_reg_C | OmpR | Cells envelop protein folding |
| WP_001253818.1/WP_001157751.1 | EnvZ/OmpR | + | 2TM-HAMP-HisKA-HATPase_c | REC-Trans_reg_C | OmpR | Osmosis regulation |
| WP_001212189.1/WP_000697899.1 | BasS/BasR | + | 2TM-HAMP-HisKA-HATPase_c | REC-Trans_reg_C | OmpR | Modification of lipopolysaccharide |
| WP_000870073.1/WP_000137854.1 | BaeS/BaeR | _ | TM-HAMP-HisKA-HATPase_c | REC-Trans_reg_C | OmpR | Multidrug efflux |
| WP_000732946.1/WP_001080048.1 | RstB/RstA | _ | 2TM-HAMP-HisKA-HATPase_c | REC-Trans_reg_C | OmpR | Capsular synthesis |
| WP_000146192.1/WP_001188783.1 | GlnL/GlnG | + | PAS-HisKA-HATPase_c | REC-AAA-HTH_8 | NtrC | Response to limitation of nitrogen |
| WP_000779338.1/WP_001221574.1 | QseC/QseB | _ | 2CSK_N-HisKA-HATPase_c | REC-Trans_reg_C | OmpR | Regulation of flagella and motility genes |
| WP_001676035.1/WP_000625591.1 | GlrK/GlrR | + | TM-HisKA-HATPase_c | REC-AAA/Sigma-54 | NtrC | Maintain cell envelope homeostasis |
| WP_001219533.1/WP_001187046.1 | CreC/CreB | _ | TM-sCache_3_2-HAMP-HisKA-HATPase_c | REC-Trans_reg_C | OmpR | Regulation of catabolite, motility |
| WP_001214411.1/WP_000190927.1 | TtrS/TtrR | + | Phosphonate-bd-TM-HisKA-HATPase_c | REC-HTH_LUXR | LuxR | Tetrathionate respiration |
| WP_000872348.1/WP_001237934.1 | TctE/TctD | + | 2CSK_N-TM-HAMP-HisKA-HATPase_c | REC-Trans_reg_C | OmpR | Uptake of tricarboxylic acids |
| WP_000678486.1/WP_000930836.1 | PgtB/PgtA | + | 2TM-HAMP-HisKA-HATPase_c | REC-Sigma54_activ_2-HTH_8 | NtrC | Phosphoglycerate transport |
| WP_000240033.1/WP_000698207.1 | YedV/YedW | + | 2TM-HAMP-HisKA-HATPase_c | REC-Trans_reg_C | OmpR | Confers resistance to copper and several drugs when induced |
| WP_001031687.1/WP_000986522.1 | PhoQ/PhoP | _ | PhoQ_Sensor-TM-HisKA-HATPase_c | REC-Trans_reg_C | OmpR | Mg^2+^ starvation and virulence |
| WP_000272845.1/WP_000598637.1 | YehU/YehT | + | TM-5TM_5TMR_LYT-GAF-His_kinase  -HATPase_c | REC-LytTR | LytTR | part of a nutrient-sensing network |
| WP_000682894.1/WP_000611307.1 | DcuS/DcuR | + | 2TM-sCache_3_2-PAS-SPOB_a-HATPase_c | REC | CitB | Controls the expression of genes of C(4)-dicarboxylate metabolism |
| WP_000121823.1/WP_000138786.1 | DpiB/DpiA | + | sCache_3_2-TM-PAS-SPOB_a-HATPase_c | REC-CitT | CitB | Regulate the citrate fermentation, and inducing the SOS response |
| WP_001677995.1/WP_000377406.1 | CitA/CitB | + | TM- sCache_3_2-PAS-HATPase_c | REC-CitT | CitB | Citrate uptake and metabolism |

The English codes represent the corresponding domains of Pfam and Smart；TM, transmembrane domain.
